# MultiK: an automated tool to determine optimal cluster numbers in single-cell RNA sequencing data

**DOI:** 10.1186/s13059-021-02445-5

**Published:** 2021-08-19

**Authors:** Siyao Liu, Aatish Thennavan, Joseph P. Garay, J. S. Marron, Charles M. Perou

**Affiliations:** 1 Lineberger Comprehensive Cancer Center, University of North Carolina at Chapel Hill, Marsico Hall, 5th floor, CB#7599, 125 Mason Farm Road, Chapel Hill, NC 27599 USA; 2 Department of Genetics, School of Medicine, University of North Carolina at Chapel Hill, Chapel Hill, NC 27599 USA; 3 Curriculum in Bioinformatics and Computational Biology, University of North Carolina, Chapel Hill, NC 27599 USA; 4 Oral and Craniofacial Biomedicine Program, School of Dentistry, University of North Carolina at Chapel Hill, Chapel Hill, NC 27599 USA; 5 Department of Surgery, Oregon Health & Science University, Portland, OR 97239 USA; 6 Department of Statistics and Operation Research, University of North Carolina at Chapel Hill, 352 Hanes Hall CB#3260, Chapel Hill, NC 27599 USA; 7 Department of Pathology and Laboratory Medicine, University of North Carolina at Chapel Hill, Chapel Hill, NC 27599 USA

**Keywords:** Single-cell RNA-seq, Clustering, Multi-scale, Multi-resolution, Genomics, Reproducibility

## Abstract

Single-cell RNA sequencing (scRNA-seq) provides new opportunities to characterize cell populations, typically accomplished through some type of clustering analysis. Estimation of the optimal cluster number (*K*) is a crucial step but often ignored. Our approach improves most current scRNA-seq cluster methods by providing an objective estimation of the number of groups using a multi-resolution perspective. MultiK is a tool for objective selection of insightful *Ks* and achieves high robustness through a consensus clustering approach. We demonstrate that MultiK identifies reproducible groups in scRNA-seq data, thus providing an objective means to estimating the number of possible groups or cell-type populations present.

**Supplementary Information:**

The online version contains supplementary material available at 10.1186/s13059-021-02445-5.

## Background

Single-cell RNA sequencing (scRNA-seq) has emerged as a powerful tool for gene expression profiling, offering opportunities to study individuals cells and features like cell-to-cell variability and intra-tumor heterogeneity at a new level of data resolution [1–4]. Identifying distinct groups of cells based on transcriptome similarity, often called “clustering,” has been a key component in scRNA-seq analysis. Accurate and reliable characterization of cell types and/or cell states in healthy and diseased tissues such as cancers not only provides fundamental insights into disease development and progression, but also provides enlightenment into therapeutic resistance, which has important implications for guiding treatment [5–7].

Estimating the optimal number of data-driven clusters (henceforth called “*K*”) is a critical issue in clustering analysis. Some clustering algorithms, such as *K*-means, require an initial choice of *K*. Other algorithms such as hierarchical clustering allow choosing *K* after partitioning. Because the cluster numbers are unknown a priori, but need to be discovered from the data, the choice of a starting *K* is typically subjective, with interpretations largely relying on the user’s desired clustering resolution and/or prior knowledge.

One approach to determining an optimal *K* is to use validation indices that evaluate the results of a clustering procedure for different values of *K*. Many indices have been proposed for this purpose, such as the elbow method [8], the silhouette index [9], the GAP statistic [10], Clest [11], and prediction strength [12]. The main drawback of these approaches is that they lack a “multi-resolution/multi-scale” clustering perspective. A single clustering resolution approach hinders the user’s ability to explore clusters at different scales, which could also be biologically relevant. Several multi-resolution methods were recently proposed for choosing *K* in scRNA-seq data, such as Clustree [13], scClustViz [14], IKAP [15], and TooManyCells [16]. However, most of these were designed to explore different choices of *K* by visualizing the clusters at different resolutions, and do not explicitly inform the choice of *K*.

Previous work has shown that clustering of perturbed data multiple times at various clustering resolutions can inform the choice of *K* [17]. The main idea is that the optimal cluster number should be stable to small perturbations such as resampling, and therefore, the stability of clusters at different resolutions can be measured to infer optimal *K*. Monti et al. proposed “Consensus Clustering” [18, 19], but it was not scalable for large-scale scRNA-seq data. Single-cell consensus clustering (SC3) [20] was recently developed for that case and uses a consensus clustering framework that repeatedly applies *K*-means clustering to find the consensus. The estimation of *K* was based on Tracy-Widom theory [21]; however, previous studies have shown that the accuracy of SC3 is sensitive to the parameter used in dimension reduction and transformation prior to clustering and tends to overestimate *K* [22]. Hence, there is still a need to develop novel automated tools for objective estimation of *K* in scRNA-seq data contexts.

Here, we develop such a data-driven tool, termed “MultiK,” which objectively selects multiple insightful numbers of clusters from the data. We hypothesize that there exist different levels of cluster resolution (i.e., multi-resolution) that are biologically relevant in the data: some clusters are more distinct (e.g., cell types), and others are less distinct but still different (such as related subtypes within a common cell type). MultiK presents multiple diagnostic plots to assist in the determination of meaningful *Ks* in the data and makes objective optimal *K* suggestions, which encompasses both high- and low-resolution parameters.

## Results

### Overview of MultiK workflow

**Fig. 1:**
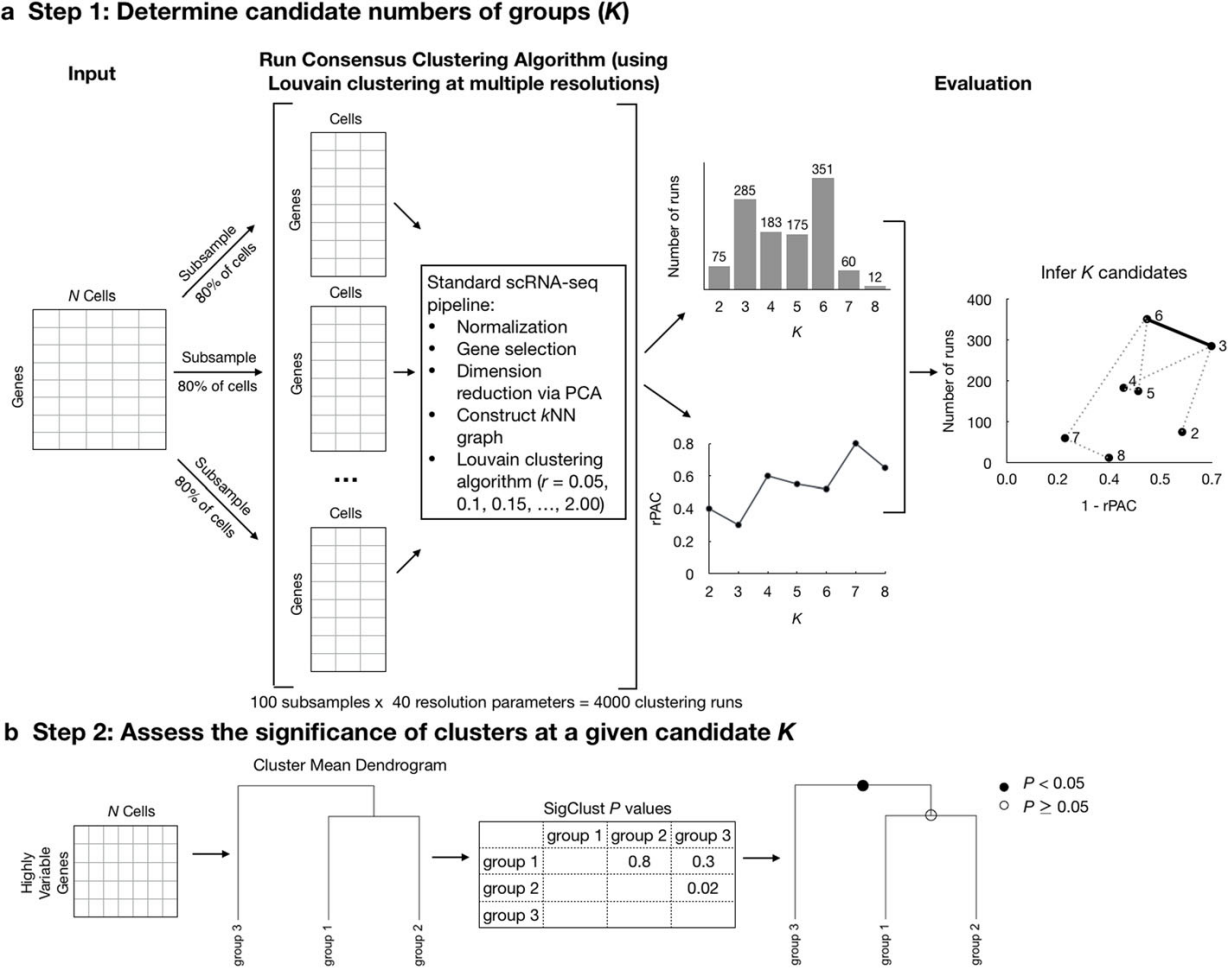
Overview of MultiK workflow. **a** Step 1: Starting from a count matrix, MultiK subsamples 80% of the cells and performs a standard scRNA-seq pipeline using Seurat (version 3.1.2) 100 times over each of 40 resolution parameters (from 0.05 to 2 with an increment of 0.05). Then, MultiK determines candidate *Ks* based on the frequency of *K* and rPAC across all subsampling runs. **b** Step 2: MultiK assigns class or subclass labels to clusters at candidate *K* levels. MultiK first constructs a dendrogram of the cluster centroids using hierarchical clustering and then runs SigClust on each pair of terminal clusters to determine classes and subclasses

The MultiK analysis workflow includes two main steps (Fig. 1). First, in the spirit of consensus clustering, MultiK determines candidate numbers of clusters via subsampling 80% of the cells from the original complete dataset. This is done across 100 runs of the Louvain clustering (as implemented in Seurat) over each of 40 resolution parameters (from 0.05 to 2 with an increment of 0.05). During each run, features are re-selected each time to cluster the cells. Then, for each *K*, MultiK aggregates all the clustering runs that give rise to the same *K* groups regardless of the resolution parameter and computes a consensus matrix. MultiK then evaluates the consensus of clustering using two metrics: (1) for each *K*, the frequency of runs where that *K* is observed (Fig. 1a center), and (2) the relative proportion of ambiguous clustering PAC (rPAC) score for each *K* (Fig. 1a center**)**, which is a variation of the PAC score [23]. PAC quantifies the proportion of entries in the consensus matrix strictly between the lower and upper bounds that determine ambiguity. The rPAC criterion addresses the upward bias of PAC towards higher *K* by better handling the proportion of zeros in the consensus matrix. Combining both measures, MultiK produces a scatter plot that shows the relationship between the frequency of *K* and (1 – rPAC) for each observed *K* (Fig. 1a right). To determine several multi-scale optimal *K* candidates (mostly 2 and up to 3), MultiK applies a convex hull approach [24]. This is based on the upper right of the smallest convex polygon that encloses all the points. MultiK takes extreme points from this set and uses a frequency cutoff of 100 to select candidate *Ks*.

Once candidate *Ks* are determined, MultiK then performs a second step: label each cluster as either a *class* or *subclass* using Statistical Significance of Clustering (SigClust) [25, 26] (Fig. 1b). MultiK first constructs a dendrogram of the cluster centroids using hierarchical clustering. Then, MultiK runs SigClust on each pair of terminal clusters. Significant terminal pairs in the dendrogram determine classes, and non-significant pairs are subclasses. For consistency of the whole dendrogram, when any split is significant, all parent splits are also considered to be significant. In this way, MultiK assigns class and subclass labels to each terminal cluster (i.e., the leaves of the dendrogram) based on the SigClust significance. This assessment of cluster significance, after deciding on the value of optimal *K*, helps elucidate the structural relationships between the identified clusters as well.

### Performance on a demonstration dataset

**Fig. 2:**
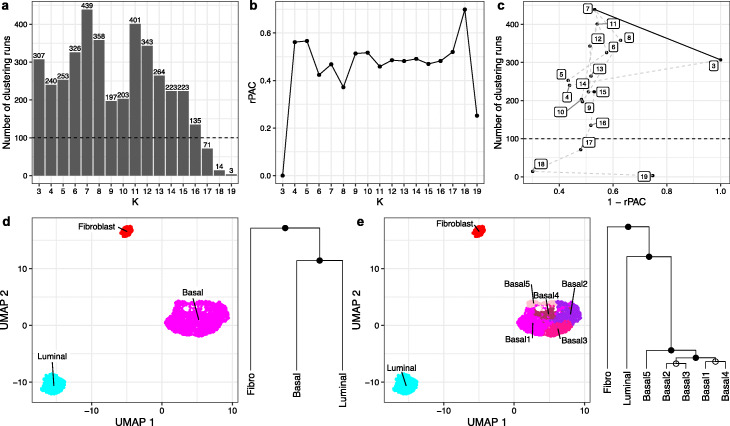
MultiK identified *K* = 3 and 7 as optimal in the 3 cell line mixture dataset. **a** Bar plot of the frequency of runs for each *K* across a total of 4000 subsampling runs. The numbers above each vertical bar represent the frequency of runs. The dotted line is the frequency 100 threshold. **b** Plot of relative PAC score for each *K*. The *X*-axis represents *K*. The *Y*-axis represents the relative PAC score. **c** Scatterplot of (1 - rPAC) and the frequency of *K*. Each dot represents a level of *K* from **a** and **b**. The thin dotted line connects each *K* to show the ordering. The fat solid line connects the candidates for optimal *K*. **d**, **e** Left is 2-d UMAP plot of the Louvain clustering from Seurat (version 3.1.2) on the full data matrix at the suggested candidate *K* level. Each dot represents a single cell and each color represents a cluster at a given *K* level. Right is the pairwise SigClust result mapped onto the cluster mean dendrogram at suggested candidate *K* levels. Closed circles on the nodes indicate significant SigClust *p* values (< 0.05) suggesting a cluster is a class, whereas open circles on the nodes indicate non-significant SigClust *p* values (≥ 0.05) suggesting a cluster is a subclass. Fibro=fibroblast

We used a dataset generated from a mixture of 3 cell lines grown in vitro [27] to first evaluate MultiK’s performance. The 3 cell line mixture dataset consists of single cells from 3 distinct cell lines (human dermal fibroblasts-skin, breast cancer luminal epithelial cell line MCF-7, and breast cancer basal-like epithelial cell line MDA-MB-468) in a 1:3:6 ratio. The MultiK diagnostic plots (Fig. 2a–c) reveal that *K* = 3 and 7 are the optimal solutions. At the low-resolution level, MultiK identifies *K* = 3 classes, corresponding to the 3 cell lines (Additional file 1: Fig. S1a and Additional file 2: Table S1). At the high-resolution level, MultiK finds *K* = 7, with 4 classes, one of which has 4 subclasses (Fig. 2d, e). Further differential gene expression analysis (Additional file 1: Fig. S1b and Additional file 2: Table S1) reveals that the four classes are fibroblast (COL1A1, VIM), luminal (GATA3, AREG), basal (KRT7, KRT17), and basal/claudin-low; among the 4 subclasses, 3 are basal cell line groups that reflect different phases of cell cycle, and the last one is the typical basal cell line feature group.

### Synthetic dataset experiments

To further validate our method, we carried out two sets of synthetic data set experiments (see the “Methods” section details). In the first method, we randomly took a group of luminal cells and knowingly perturbed a specific group of genes in the 3 cell line mixture data, thereby providing a true known gold standard fourth group. The amount of perturbation was done through either varying the Poisson parameter that was used to model the gene counts or varying the number of modified genes. The results from MultiK in the low-resolution space under each perturbation are shown in the Uniform Manifold Approximation and Projection (UMAP) plots in Additional file 1: Fig. S2. The first row in this figure shows that when 1 is the Poisson parameter, 100 modified genes are needed for MultiK to identify the synthetic cluster as a subclass, and 350 modified genes are needed to identify it as a class. However, as the Poisson parameter increases to 2 (the second row), only 60 and 100 modified genes were needed to identify the synthetic cluster as a subclass and a class respectively by MultiK. And when the Poisson parameter increases to 4 (the third row), even fewer modified genes were needed to see the same phenomenon.

To benchmark MultiK, we considered three other methods: Seurat (the Louvain clustering at the default resolution parameter 0.8) [28], SC3 [20], and IKAP [15]. Consistent with previous findings [22], we found that SC3 overestimated the number of groups, and Seurat roughly corresponded with MultiK high-resolution but was less stable (Additional file 2: Table S2). Although IKAP produced similar results as MultiK low-resolution, it was not as sensitive as MultiK in that IKAP did not detect the synthetic group until a stronger signal was introduced, whereas MultiK was able to identify the synthetic group with a weaker signal (either fewer modified genes or lower Poisson parameter added to the data).

For the second set of synthetic experiments, we simulated 3 scRNA-seq datasets with known groups using Splatter package [29], and then tested the performance of MultiK. The first dataset had 2 equal groups (with 500 cells in each group); the second dataset had 3 unequal groups (with group probabilities 0.1, 0.3, and 0.6); the third dataset had 5 equal groups (with 200 cells in each group). Visual inspection of PCA projections showed distinct separation of the simulated groups (left column in Additional file 1: Fig. S3), except in the 5 equal group data set where 3 of the 5 groups were clustered together, which could be due to the number of PC projections being displayed here. As expected, in each dataset, MultiK successfully identified the expected number of groups in the low *K* space, and the cell labels perfectly matched the group labels (right columns in Additional file 1: Fig. S3).

### Identification of rare cell populations

Detection of rare cell type has been a challenging and important task in scRNA-seq analysis. To specifically evaluate the sensitivity of MultiK in identifying rare cell populations, we performed another simulation experiment using our 3 cell line mixture dataset as follows: we systematically removed different percentages of the smallest group (fibroblast, present at 8%) in the 3 cell line mixture dataset and ran MultiK. Additional file 1: Fig. S4a shows the number of clusters MultiK identified as a function of the percentage of the fibroblast cells in the total number of cells. MultiK accurately identified the rare cell population even when only 0.5% of the cells (*N* = 13) were fibroblasts. Additional file 1: Fig. S4b, c shows the MultiK diagnostic plots for 1% and 2% of the fibroblast cells. In all the cases tried, most of these were like the results in the 1% case, in which MultiK identified 3 and 7 as optimal *K*s, and when *K* = 3, the cell labels from MultiK perfectly matched the true cell labels.

### Application to normal mouse mammary gland datasets

**Fig. 3:**
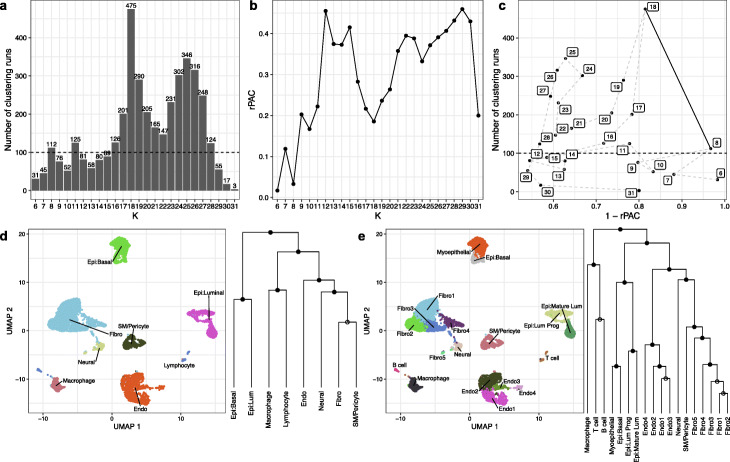
MultiK identified *K* = 8 and 18 as optimal in the FVB3-mammary dataset. **a** Bar plot of the frequency of runs for each *K* across a total of 4000 subsampling runs. The numbers above each vertical bar represent the frequency of runs. The dotted line is the frequency 100 threshold. **b** Plot of relative PAC score for each *K*. The *X*-axis represents *K*. The *Y*-axis represents the relative PAC score. **c** Scatterplot of (1 - rPAC) and the frequency of runs for each *K*. Each dot represents a level of *K* from **a** and **b**. The thin dotted line connects each *K* to show the ordering. The fat solid line connects the candidates for optimal *K*. **d**, **e** Left is 2-d UMAP plot of the Louvain clustering from Seurat (version 3.1.2) on the full data matrix at the suggested candidate *K* levels. Each dot represents a single cell and each color represents a cluster at a given *K* level. Right is the pairwise SigClust result mapped onto the cluster mean dendrogram at suggested candidate *K* levels. Closed circles on the nodes indicate significant SigClust *p* values (< 0.05) suggesting a cluster is a class, whereas open circles on the nodes indicate non-significant SigClust *p* values (≥ 0.05) suggesting a cluster is a subclass. Fibro=fibroblast, Epi=epithelial, Endo=endothelial, SM=smooth muscle, Lum Pro=luminal progenitor, Mature Lum=mature luminal

To test whether MultiK can produce robust clustering solutions using complex tissue specimens, we performed MultiK on five normal mouse mammary gland datasets (see the “Methods” section for detailed dataset description and Additional file 2: Table S3), and which also spanned two sequencing technologies. MultiK suggested *K* = 8 and 18 as optimal in the FVB3-mammary gland set (Fig. 3a–c). When *K* = 8, MultiK identified 8 clusters including 2 epithelial clusters (luminal [Cd24a, Krt8, Krt18] and basal cluster [Acta2, Krt14, Krt17]), 2 immune clusters (macrophage [Fcer1g, Cd68] and lymphocyte [Cd74]), 1 fibroblast cluster [Dcn, Col3a1], 1 endothelial cluster [Fabp4, Cldn5], 1 smooth muscle/pericyte cluster [Procr], and 1 neural cluster [Mpz, Mbp] (Fig. 3d). All clusters were assigned as classes except that the fibroblast cluster and the smooth muscle/pericyte cluster were assigned as subclasses. The similarity in their top differentially expressed genes between these two clusters is shown in Additional file 1: Fig. S5a and Additional file 2: Table S4. When *K* = 18, more classes and subclasses were identified (Fig. 3e). The luminal epithelial cluster was split into 2 classes: mature luminal and luminal progenitor. The basal epithelial cluster was also split into 2 classes: basal and myoepithelial. Additionally, MultiK found 5 fibroblast clusters, but only 3 were assigned to classes: one showing a typical fibroblast phenotype, another associated with cell stress sensing/apoptosis, and the third related to cell motility and migration. A similar finding was seen in the endothelial cells as well: 3 classes were identified from 4 endothelial groups, one showing typical endothelial features, another related to cell stress/apoptosis, and the third showing a lymphatic phenotype. The lymphocyte cluster was split into B and T cell clusters, but interestingly, they were assigned to subclasses (Additional file 1: Fig. S5b).

Next, we assessed the reproducibility (defined as an ability of a group to be found in multiple independent data sets) of these identified groups across datasets. We applied MultiK to the rest of the normal mouse mammary gland datasets and identified low *K* and high *K* solutions (related MultiK diagnostic plots are provided in Additional file 1: Fig. S6, and differentially expressed gene lists for each cluster are provided in Additional file 2: Tables S4-S8). Then, we performed gene set enrichment analysis [30, 31] in both the low-resolution and high-resolution spaces (see the “Methods” section details, Additional file 1: Fig. S7a, b). A group was considered reproducible if it was present in at least 3 out of the 4 normal mouse mammary gland datasets tested. Using the FVB3-mammary dataset as a reference set, we found that all 8 groups in the low-resolution space were present in the other datasets except for the neural group, which was present in only 2 of the 4 datasets (Fig. 5a). The two subclasses (fibroblast and smooth muscle/pericytes) were also repeatedly identified in the other datasets. In the high-resolution space, 13 out of the 18 groups were reproducibly identified including 6 subclasses (2 fibroblast subclasses, 2 endothelial subclasses, and 2 lymphocyte subclasses) (Fig. 5b). Combining both low-resolution and high-resolution, we identified a total of 15 reproducible groups in the normal mouse mammary gland data (Additional file 2: Table S16).

### Application to human T cell datasets

Recent single-cell studies characterizing T cells by gene expression analyses have defined multiple known, and several novel, T cell subsets associated with response and resistance to immunotherapies [32–37]. However, the nomenclature used to characterize these T cell subsets and/or states varies across studies, and few if any did across study comparisons. Importantly, there is little consensus on the number and nature of these T cell groups across data sets, and how they might be validated in independent studies. To identify reproducible T cell groups, and further test the performance of MultiK, we collected 6 public human scRNA-seq T cell datasets spanning multiple cancer types and applied MultiK, together with gene set enrichment analysis (see the “Methods” section details, Additional file 1: Figs. S7c, d, S8, and Additional file 2: Tables S3, S9-S14). Because the Azizi et al. breast cancer dataset contained the largest number of cells (~27,000), we used it as our reference set and then compared it to the other 5 datasets. In the low-resolution space (*K* = 13), MultiK identified 6 reproducible T cell groups including 2 regulatory T cell groups (Treg 1, Treg 2), 1 CD8 T naïve group, 1 CD8 T effector memory (CD8 Tem 2), 1 CD8 T exhausted group (CD8 Tex), and 1 CD4 T naïve group (Fig. 5). In the high-resolution space (*K* = 27), MultiK identified 6 additional reproducible groups including 3 CD8 T central memory subsets (CD8 Tcm 1.1, CD8 Tcm 1.2, CD8 Tcm 1.3), 1 CD8 T tissue-resident memory subset (CD8 Trm 2), and 2 CD4 follicular helper subsets (CD4 Tfh 1.2, CD4 Tfh 2.1) (Additional file 1: Figs. S7d, S9); thus, in total, our MultiK analysis identified 12 groups (5 classes and 7 subclasses) of T cells as being present across multiple scRNA-seq data sets (Additional file 2: Table S17).

Multiple studies have provided evidence that many of these T cell subsets play crucial roles in modulating cancer immunosurveillance and are the key targets of modulation by immune checkpoint inhibition [32, 33, 38]. For example, two studies in patients with non-small cell lung cancer (NSCLC) and triple-negative breast cancer (TNBC) have described the presence of CD8 Trm cells expressing integrin αE (ITGAE, encoding CD103) [32], and Savas et al. further found that the gene signature of the CD8 Trm subset significantly correlated with better survival in patients. Guo et al. [33] found two subsets of Tregs in the lung tumor microenvironment, distinguished by high or low expression of tumor necrosis factor receptor superfamily member 9 (TNFRSF9, encoding 4-1BB); they showed that the activated tumor Treg subset (expressing high levels of immune checkpoints) was associated with poor prognosis in NSCLC, which was further supported by a recent independent study of bladder tumors [37].

To assess whether our newly derived reproducible T cell groups provide any prognostic value for predicting breast cancer patient outcomes, we developed gene expression signatures (see the “Methods” section) for each of the 12 reproducible T cell groups found here and applied these signatures to 5 breast cancer bulk tumor gene expression data sets, in which associated clinical information was available: the Molecular Taxonomy of Breast Cancer International Consortium (METABRIC) [39], the Harrell 855 set [40] (855 primary breast tumors combined from four public microarray studies), the Cancer Genome Atlas breast tumors (TCGA BRCA) [41], the Sweden Cancerome Analysis Network-Breast (SCAN-B) [42], and CALGB 40601 [43] (a HER2+ neoadjuvant trial). To compare with our new T cell signatures, we also evaluated additional gene expression features including single gene expression values of MKI67, CD274 (PD-L1), ERBB2, and the expression signatures that are a B cell/T cell cooperation signature [38], an Immunoglobulin G (IGG) signature [44], and seven CIBERSORT LM22 T cell signatures [45]. We performed the signature analysis on all data sets combined and then stratified into the 3 clinical groups (ER+HER2−, HER2+, and TNBC based on immunohistochemistry (IHC) status), thus creating 4 main data sets: (1) all samples from the METABRIC, Harrell 855, TCGA BRCA, and SCAN-B; (2) ER+HER2− samples from the METABRIC, Harrell 855, TCGA BRCA, and SCAN-B; (3) HER2+ samples from the Harrell 855, TCGA BRCA, SCAN-B, and CALGB 40601; (4) TNBC samples from the METABRIC, Harrell 855, TCGA BRCA, and SCAN-B. We also performed the analysis on each data set individually, on all patients as well as within stratified clinical groups.

We found that 6 out of the 12 repeatedly identifiable T cell signatures identified from MultiK were significantly associated with improved overall survival in at least three of the combined sets (Fig. 6): Treg 1, CD8 Tem 2, CD4 naïve, and CD8 naïve were significant in all 4 sets; CD8 Tcm 1.1 and CD8 Tcm 1.3 were significant in all the combined sets except for the ER+HER2− set. In particular, we found that the CD4 T naïve signature was significantly associated with overall survival in all sets, supporting previous evidence for a role of intra-tumoral CD4 T cells from both mouse and human studies [46, 47]. We also found that the two CD4 T follicular helper subsets significantly correlated with survival in TNBC samples, which was in line with previous findings from our group that CD4 T follicular helper cells mediate immune checkpoint response using mouse models of TNBC [38]. Furthermore, consistent with the previous finding that the CD8 Trm signature associated with good prognosis in TNBC [32], we found that our CD8T resident memory signature was significant in the HER2+ and TNBC sets. Next, we found that one of the Treg signatures was significantly correlated with overall survival in all 4 sets, but the other Treg signature was only significant in the HER2+ and TNBC sets, suggesting that different Treg subsets may play different roles in breast cancer subtypes. Lastly, in Additional file 1: Fig. S10, we show the results of similar analyses, but performed on each individual data set according to clinical receptor subtypes.

### Discussion

scRNA-seq is a powerful new approach for studying the transcriptomes of cell lines, tissues, tumors, and diseased states. It is being widely used to identify what are often claimed to be novel cell types; however, the analysis methods are typically complex, and the user is often simply given a visual representation of the data with no assessment of the robustness of the groupings. That is, there is no data-driven guidance on how many cell populations/types are present. To address this need, we developed MultiK, an automated “multi-resolution” approach that objectively selects multiple insightful numbers of clusters (*K*) in scRNA-seq data. We demonstrated that MultiK successfully identified *K* in a ground truth dataset and was sensitive in the identification of classes and subclasses in a synthetic “spike in” experiment as well as additional simulated datasets. We further applied MultiK to identify reproducible groups in complex tissue datasets, including mouse mammary glands and multiple T cell data sets. In both cases, we identified most of the previously known subsets/cell populations and did so without any prior biological knowledge of true clusters present in the data.

**Fig. 4:**
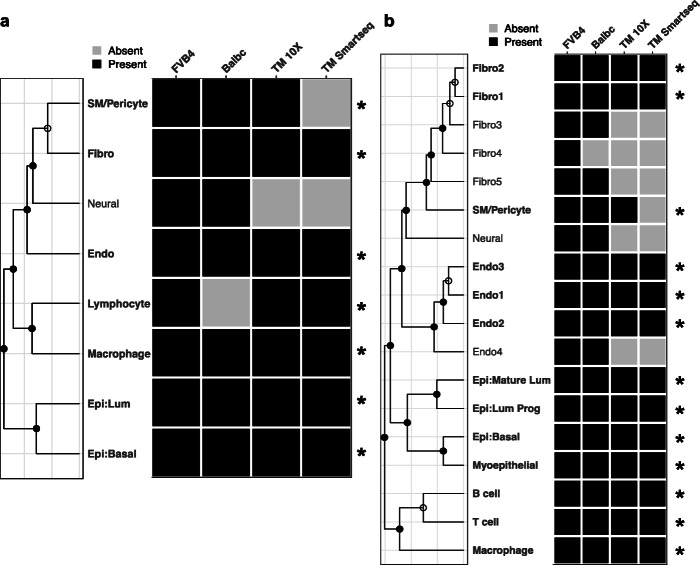
MultiK identified robust and reproducible groups across five mouse mammary gland datasets via gene set enrichment analysis. Using the FVB3-mammary dataset as reference, gene set analysis across datasets in both low-resolution (**a**) and high-resolution (**b**) space after applying MultiK. In each panel, left is a dendrogram constructed from the cluster means in the FVB3-mammary dataset. Closed circles on the nodes indicate significant SigClust *p* values (< 0.05) suggesting a cluster is a class, whereas open circles on the nodes indicate non-significant SigClust *p* values (≥ 0.05) suggesting a cluster is a subclass. Right is a heatmap showing the presence (colored black) or absence (colored gray) of each FVB3-mammary group in the other datasets. The reproducible groups are highlighted with asterisks at the end of each row in the heatmap

**Fig. 5:**
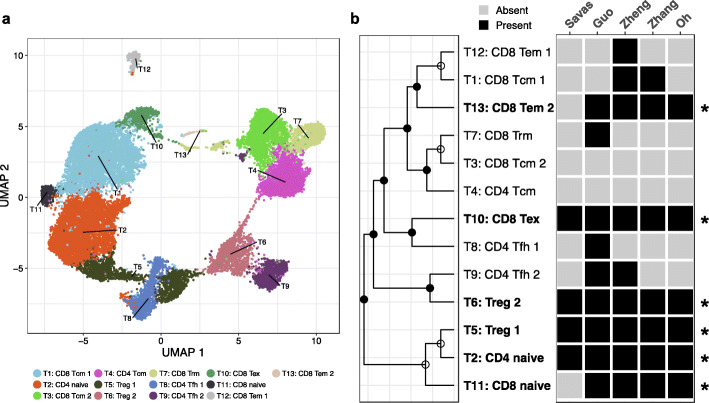
MultiK identified robust and reproducible groups in the low-resolution space across six human T cell datasets via gene set enrichment analysis. **a** 2-d UMAP plot of the clusters at low-resolution (*K* = 13) in the Azizi et al. dataset. Each dot represents a single cell and each color presents a cluster. **b** Heatmap of reproducible T cell groups found in the other dataset using gene set enrichment analysis. Left is a dendrogram constructed from the 13 cluster means in the Azizi et al. dataset. Closed circles on the nodes indicate significant SigClust *p* values (< 0.05) suggesting a cluster is a class, whereas open circles on the nodes indicate non-significant SigClust *p* values (≥0.05) suggesting a cluster is a subclass. Right is a heatmap showing the presence (colored black) or absence (colored gray) of each Azizi et al. group in the other datasets. The reproducible groups are highlighted with asterisks at the end of each row in the heatmap

Given the diversity and abundance of scRNA-seq data available in the public domain, we also observed that MultiK is robust to different single-cell sequencing technologies. For example, in our real dataset applications (both mouse mammary gland and human T cell datasets), three of which were Smart-seq2 and the rest were 10X genomics sequencing technologies (see Additional file 2: Table S3), we found the same reproducible groups across both platforms in our presented studies. Specifically, in the mouse mammary gland case, we found 6/7 reproducible groups on both 10X and Smart-seq2 platforms in the low *K* space, and all 8 reproducible groups on both platforms in the high *K* space (Fig. 4, Additional file 2: Table S15-16). In the human T cell case, all the reproducible groups were detected by both platforms in both low *K* and high *K* space (Fig. 5, Additional file 1: Figs. S7c, d, S9, Additional file 2: Tables S15, S17).

Our proposed tool has several merits. First, to our best knowledge, this is the first tool that provides guidance on a multi-scale choice of *K* in scRNA-seq data. There are a number of sophisticated clustering and visualization tools developed in the single cell domain, including BackSPIN [48], SCCAF [49], and scGNN [50]. However, none of these methods suggests what is the optimal number(s) of clusters/groups present in the data. The choice of *K* often relies on the scientist to eyeball how many “groups” they think are present. The main contribution of our study is to go beyond this previous work that focused on clustering and the goal of MultiK is to provide an objective assessment of *K*. Importantly, MultiK benefits most clustering methods because many methods may need an input value of *K* to even begin a clustering analysis. In these cases, we recommend running MultiK first, which will give a rigorous choice of *K* for use as input to any preferred clustering method of interest that could be run next. For methods that do not need an input choice of *K*, but which do not provide outputs on the optimal *K*, MultiK provides such a data-driven suggestion for optimal *K* in both the low-resolution space and high-resolution space. Other tools such as Clustree, TooManyCells, and scClustViz serve as visualization tools for exploring clusters at different clustering resolutions in scRNA-seq data, but they do not provide data-driven guidance on the choice of *K*. While IKAP informs *K* by setting an initial *K*_*max*_ and iteratively merging the 2 nearest clusters to obtain optimal *K* assessed by the GAP statistic, it is a single resolution approach and thus only selects a single option. It is clear from our multi-scale approach, that suggested *K*s at multiple levels of resolution are both biologically helpful, and objectively derived from the data. Next, MultiK can identify robust clustering patterns or structures that are sensitive to relatively weak signals and produce consistent and stable clustering across datasets and across scRNA-seq platforms. Lastly, MultiK can investigate the structural relationships between the clusters after the optimal *K* is chosen. Further assignment of classes and subclasses provides biological insights and interpretation of the clustering structure in the data.

We benchmarked the performance of MultiK against several alternatives including IKAP, SC3, and Seurat (default resolution parameter 0.8) in a synthetic experiment and demonstrated that MultiK gave sensitive and stable identification of classes and subclasses relative to other methods. To further demonstrate MultiK’s ability in finding reproducible clusters, we applied MultiK to multiple complex tissue datasets in both mouse and human. We showed that MultiK identified most of the known groups in normal mouse mammary glands, providing biological validation of the useful application of MultiK. In the human T cell case, MultiK identified a total of 12 reproducible T cell subsets spanning 6 different data sets that represent multiple cancer types. Moreover, consistent with previous findings [32, 33, 37], some of these reproducible T cell signatures showed prognostic values in predicting breast cancer patient’s survival. These T cell data set findings are especially important because as seen in the literature, most previous analyses likely overestimated the number of reproducible T cell subsets in the tumor immune microenvironment, which may lead to irreproducible findings across studies.

Despite the advantages of MultiK, we note two limitations to our method. First, our approach estimates *K* based on repeatedly perturbing the original data via subsampling. Small, rare clusters may not be detected due to random sampling variation, and it is possible that the sampled distribution may differ significantly from the null distribution; however, in our rare cell populations analyses where we intentionally created a rare group, we were able to detect this rare group down to the 0.5% level. Second, MultiK is computationally expensive for large datasets in large part due to the 100-fold subsampling and reclustering; however, multi-core ability and parallel computing can mitigate this problem.

In summary, we demonstrate that MultiK provides a novel tool that finds meaningful multi-resolution optimal cluster numbers, so-called *K*, in scRNA-seq data and provides stable clustering solutions across datasets. In addition, it is worth noting that the concept of MultiK is applicable beyond scRNA-seq data. In the future, MultiK can also be tailored and extended to other data types, such as scATACseq and mass cytometry data.

## Methods

### Datasets

We used 12 scRNA-seq datasets to benchmark our method (Additional file 2: Table S3). The first dataset was a 3 cell line mixture generated in our lab [27]; for this dataset, we have two pieces of information providing ground truth cell identities: (1) the mixing ratio of the cell lines (1:3:6) and (2) SNPs discovered from bulk RNA-seq data unique to each cell line. The second, third, and fourth datasets were derived from normal adult whole mouse mammary glands of 2 mouse strains with different genetic backgrounds (FVB/NJ mouse strain: FVB3-mammary, FVB4-mammary, and Balbc mouse strain: Balbc-mammary). The exact cell labels in these datasets were unknown; however, we can use biological knowledge to determine the cell types as this normal tissue type has been well studied [51–56]. The fifth and sixth datasets were normal adult mouse mammary gland datasets of C57BL/6 mouse strain from the Tabula Muris consortium [57] (referred to as TM 10X-mammary and TM Smartseq-mammary, respectively). The rest of the datasets (sets 7–12) were human T lymphocyte datasets from various cancer cohorts: 2 in breast cancer [32, 35], 1 in lung cancer [33], 1 in liver cancer [34], 1 in colorectal cancer [36], and 1 in bladder cancer [37].

### Data preprocessing

For the mixture of 3 human cell lines and the normal mouse mammary gland datasets, genes that have zero count in all cells were discarded. Cells were removed if the number of total counts or the number of detected genes (count larger than zero) or the proportion of expressed mitochondrial genes was larger or smaller than the pre-defined thresholds, which were the medians of all cells ± 3 × median absolute deviation. That filtering should remove likely cell doublets or multiplets and low-quality cells, respectively.

For T cell datasets, we first developed a “T cell bioinformatics sorting rule” to systematically extract T cells from each individual dataset before clustering to make sure each dataset was preprocessed in a similar manner. Specifically, we used the raw count gene expression of three T cell markers: CD3D, CD3E, and CD3G to select cells that have nonzero counts in at least 2 of the 3 markers. These cells were then filtered as done for the mixture of 3 cell lines and the normal mouse mammary gland datasets.

### MultiK details

MultiK is built on top of Seurat (version 3.1.2) [28, 58] in R (version 3.5.2). First, MultiK takes a gene expression matrix as input, in which cells are the columns and genes are the rows. Each entry of the input matrix corresponds to the expression of a gene in each cell. MultiK subsamples 80% of the cells from the input preprocessed data matrix and applies the standard Seurat pipeline on the subsampled data matrix 100 times over 40 resolution parameters (from 0.05 to 2.00 with step size 0.05; thus, 4000 subsampling runs in total: 40 resolution parameters × 100 subsamples). The standard Seurat pipeline includes normalization, feature selection, PCA dimension reduction, and clustering via the Louvain algorithm [59]. Specifically, library size normalization is applied to the preprocessed matrix. This is done by taking each entry of the filtered matrix, dividing it by the total number of counts per cell, multiplying it by a scale factor of 10,000, and taking a log transformation. To identify highly variable genes, MultiK applies the variance stabilizing transformation (“vst”) method in the Seurat package and selects 2000 features for downstream clustering. Then, prior to dimension reduction, each gene is scaled to have mean 0 and variance 1 across cells. MultiK performs PCA dimension reduction on the scaled data with the previously selected variable features. To cluster the cells, MultiK first constructs a K-nearest neighbor graph (default 20 nearest neighbors, same as implemented in Seurat) in the first 30 PCs space. Then, the edge weights are refined based on the shared overlap in their local neighborhoods using the Jaccard similarity measure. The Louvain algorithm [59] is applied to iteratively cluster cells together.

For each candidate *K*, MultiK computes two evaluation metrics: (1) the frequency of each *K* solution across the 4000 clustering runs and (2) a score called the “relative Proportion of Ambiguous Clustering” (rPAC), which is an improvement of PAC [23]. To compute rPAC, MultiK first aggregates all the clustering runs that result in the same *K* across all the resolution parameters, and calculates a consensus matrix for each *K*, which is defined as “the proportion of clustering runs in which two samples are grouped together” [18]. Then, MultiK computes the Cumulative Distribution Function (CDF) curve for each consensus matrix. From the CDF, MultiK calculates the rPAC score for each *K* using the following formula:

$\mathrm{r}{\mathrm{PAC}}_k\left({\mu}_1,{\mu}_2\right)=\frac{{\mathrm{CDF}}_k\left({\mu}_2\right)-{\mathrm{CDF}}_k\left({\mu}_1\right)}{{\mathrm{CDF}}_k(0)}$, where (*μ*_1_, *μ*_2_) ∈ [0, 1], and *μ*_1_ and *μ*_2_ are usually chosen close to 0 and 1, respectively (such as 0.1 and 0.9).

### MultiK parameter setting

Different parameter settings impact the estimation of cluster number and clustering. Through exploration of the resolution parameter space, we found that a range from 0.05 to 2 was sufficient to capture the structure and substructure within the data, and therefore set it as the default in MultiK. In addition, we explored how the number of PCs affects clustering, knowing that a more heterogeneous dataset may need more PCs to discover different diverse cell types/subtypes. We noticed that there was a trade-off between an increase in the number of PCs and loss of stability in clustering: a small number of PCs did not provide enough dimensions to capture the real clustering structure in the lower dimensional space; a large number of PCs may introduce more noise, resulting in discovering unstable clusters. Our exploratory analysis suggested 30 PCs kept a good balance between capturing clustering structure and introducing noise, and thus is the default in MultiK.

### SigClust analysis

Once a candidate *K* was selected, we used SigClust [26] to assess the significance of individual clusters. SigClust was performed on every possible pair of clusters at a given candidate *K* using the cluster labels as cluster assignments, and a *p* value was calculated for each pair. We used the sample covariance estimates in SigClust due to the sparsity of the input data matrix (the median is zero) since the sample covariance estimate is conservative for fitting the null Gaussian.

To visualize the pairwise SigClust results, we first calculated the cluster mean for each cluster using the 2000 selected highly variable genes and then ran hierarchical clustering on the cluster means (Euclidean distance, and complete linkage) using the hc() function in R (version 3.5.2). Then, we mapped the SigClust *p* values onto the cluster mean dendrogram, according to the rules as follows: (1) a node is significant (*p* value < 0.05) if at least one pair is significant; (2) a parental node is significant if any of its children nodes is significant. We further assigned *class* and *subclass* to each individual cluster (i.e., the leaves of the dendrogram) based on the node significance. We defined a cluster to be a *class* if all the nodes that split the cluster are significant, and a cluster to be a *subclass* if one of the nodes that split the cluster is non-significant.

### Synthetic experiment

We randomly sampled 30% of the luminal cells (*N* = 162) and modified various numbers of genes (20, 30, 60, 100, 350) for these 162 cells by replacing their original gene counts with counts modeled by a Poisson distribution at various parameters (*λ* = 1, 2, 4). The counts generated by the Poisson process were performed using the rpois() function in R (version 3.5.2). In total, we generated 15 synthetic datasets from different combinations of Poisson parameters and numbers of modified genes and applied MultiK and other methods to these datasets. The rationale for generating such synthetic datasets using the approach described above are as follows: (i) It is difficult to simulate scRNA-seq data that mimics real-world data. Thus, we took real-world data and systematically modified just a small part of this, leaving the real-world data structure largely untouched. (ii) We already have a 3 cell line mixture dataset, in which the cell identities are known, thus providing a ground truth dataset for us to compare the performance of different methods. A simple way to augment the data is to introduce a synthetic cluster/group of cells by adding in some signals to the original data. (iii) We chose to modify the gene counts by fitting a Poisson distribution with various Poisson parameters, and this allowed us to see the impact of the signal that we purposely added in the data. It also allowed us to identify the contexts where the synthetic cluster can be identified as a class or a subclass. The reason why we chose to use a Poisson distribution is that multiple recent studies have shown that UMI count scRNA-seq data is not significantly zero-inflated and any observed zero-inflation is likely driven by the cell-type heterogeneity and biological variation [60, 61]. Particularly, Kim et al. [61] observed that the majority of the genes fit the expected Poisson curve in a homogeneous cell population, and their results strongly suggested that it is unnecessary to model any zero counts using a zero-inflated distribution. Inspired by that, we think it is appropriate to use a simple Poisson distribution to modify a small subset of genes in a subset of luminal cells in our 3 cell line mixture dataset.

### Additional synthetic datasets

We generated 3 simulated scRNA-seq datasets using the “group” method in splatSimulate() function from R/Splatter package [29]. Each dataset was simulated to contain 5000 genes and 1000 cells. The first dataset had 2 equal groups (with 500 cells in each group); the second dataset had 3 unequal groups (with group probabilities 0.1, 0.3, and 0.6); the third dataset had 5 equal groups (with 200 cells in each group). The code for generating the simulated datasets can be found in Github: https://github.com/siyao-liu/MultiK [62].

### Benchmarked methods

We benchmarked different methods including Seurat version 3.1.2 [58], SC3 [20], IKAP [15], and MultiK in terms of their performance in estimating the number of clusters in the synthetic experiment datasets. All methods were implemented following their guided tutorials (https://satijalab.org/seurat/v3.1/pbmc3k_tutorial.html, http://bioconductor.org/packages/release/bioc/vignettes/SC3/inst/doc/SC3.html, https://github.com/NHLBI-BCB/IKAP). Default parameters were used everywhere.

### Differential expression analysis

In all cases, we performed differential expression analysis on all genes that met the following criteria: (1) nonzero count in a minimum of 10% of the cells in either of the tested groups; (2) positive difference in the first tested group (i.e., genes are more highly expressed). We then applied the Wilcoxon rank-sum test to cells assigned to a cluster vs. all other cells. To identify significant differences, we considered genes with a Bonferroni-corrected *p* value ≤ 0.05 as significant genes that define a cluster.

### Gene set enrichment analysis and deriving reproducible groups across sets

We performed gene set enrichment analysis using R/GSA version 1.03.1 package [31]. We used the gene sets (top 200 positive/upregulated defining gene lists for each cluster, ranked by Bonferroni-corrected *p* values from lowest to highest) derived from the reference dataset and tested their enrichment in each cluster in the other datasets. For example, for the mammary gland datasets, the gene sets were developed from each cluster in both low- (*K* = 8) and high- (*K* = 18) resolution space in the FVB3 mammary gland dataset and applied to each cluster identified from the low- and high-resolution space in the FVB4 mammary gland (*K* = 9, 18), Balbc mammary gland (*K* = 10, 13), TM 10X (*K* = 15 used in both low and high *K* space), and TM Smartseq (*K* = 9, 18) datasets, respectively. The rule for selecting *K* in the low and high *K* space was the following: for datasets in which MultiK identified a single optimal *K* solution, the single optimal *K* was used in both low- and high-resolution analysis; for datasets in which MultiK identified 3 optimal *K* solutions, the lowest *K* was used in the low-resolution analysis; if the second lowest *K* was more than 2 above the lowest *K*, then the second lowest *K* was used in the high-resolution analysis; otherwise, the highest *K* was used in the high-resolution analysis.

A GSA score for each gene set in a cluster and its associated adjusted *p* value were calculated from 100 permutation runs. We considered a GSA score above a threshold (0.75 in the normal mouse mammary gland datasets and 0.6 in the T cell datasets) as highly enriched and used that threshold to identify common groups that were present in multiple datasets (related figures are provided in Additional file 1: Fig. S9).

### Survival analysis

**Fig. 6:**
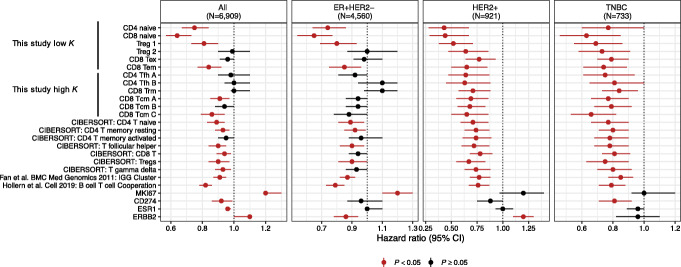
Survival analysis showed the prognostic effect of MultiK derived reproducible T cell gene signatures compared to other T cell signatures as well as single gene expression (indicated on the *Y*-axis) in external breast cancer datasets. Forest plots show hazard ratio (circles) and 95% confidence intervals (horizontal ranges) derived from the Cox proportional hazards model for overall survival in univariate analysis. Red indicates significant hazard ratio estimate (*p* value < 0.05) whereas blue indicates non-significant estimate (*p* value ≥ 0.05). The first two vertical panels show results from combining all patients (first panel) and ER+HER2− patients (second panel) from the METABRIC, Harrell 855, TCGA BRCA, and SCAN-B datasets. The third vertical panel shows results from combining HER2+ patients from the Harrell 855, TCGA BRCA, SCAN-B, and CALGB40601 datasets. The fourth vertical panel shows results from combining TNBC patients from the METABRIC, Harrell 855, TCGA BRCA, and SCAN-B datasets

The METABRIC [39], Harrell 855 set [40], TCGA BRCA [41], SCAN-B [42], and CALGB40601 [43] gene expression data were used to evaluate the prognostic value of the gene signatures derived from the reproducible T cell groups (Fig. 6, Additional file 1: Fig. S9). For CALGB40601, only the pretreated samples (*N* = 264) were used in the analysis. All gene expression data were filtered to genes that were expressed in over 70% of the samples, upper-quartile normalized and log2 transformed prior to the survival analysis. In each dataset, samples were classified into 3 clinical groups (ER+HER2−, HER2+, and TNBC) based on the clinical ER, PR, and HER2 IHC status. To make the clinical data comparable across datasets, the follow-up time was censored to 3000 days in all datasets. Overall survival was used in all datasets, except for the Harrell 855 set, where the metastasis-free survival was used (because the overall survival data was missing in that dataset). In the combined analysis, the “all” set (*N* = 6909) contained all patients from the METABRIC, Harrell 855, TCGA BRCA, and SCAN-B datasets; the “ER+HER2-” set (*N* = 4560) contained the ER+HER2− patients from the METABRIC, Harrell 855, TCGA BRCA, and SCAN-B datasets; the “HER2+” set (*N* = 921) contained HER2+ patients from the Harrell 855, TCGA BRCA, SCAN-B, and CALGB 40601 datasets (note that the HER2+ patients from the METABRIC dataset were excluded as the HER2+ patients in the METABRIC cohort did not get the trastuzumab treatment while the other sets did); the “TNBC” set (*N* = 733) contained the TNBC patients from the METABRIC, Harrell 855, TCGA BRCA, and SCAN-B datasets.

The mean expression of genes in each signature was calculated for each sample in each dataset. In the combined analysis, the mean expression of signature was further median centered within each dataset to correct for the batch effect. To determine the statistical significance, we performed univariate analysis using a Cox proportional hazards model implemented in the R/survival package by fitting the gene signature as a continuous variable. For the T cell signatures developed from this study, we identified the top 50 positive/upregulated genes for each reproducible group (genes that were significantly upregulated in the cluster as compared with other clusters with FDR < 5%) in the Azizi et al. dataset.

### Tissue processing of Balbc mammary gland and 10X chromium 3’ gene expression single-cell RNA-seq library construction and alignment

The Balbc mammary gland was harvested at 12 weeks of age and placed in 10 ml of a digestion medium containing EpiCult™-B Mouse Medium Kit (#05610, StemCell Technologies), Collagenase/Hyaluronidase (#07912, StemCell Technologies), and 1% penicillin-streptomycin (Gibco). The mammary gland was digested overnight in a thermocycler maintained at 37°C with continuous rotation. The cell pellets retrieved from these suspensions were treated with a 1:4 solution of Hanks balanced salt solution (HBSS) (Gibco) and ammonium chloride to remove the red blood cells (RBCs). After RBC removal, the cell suspensions were trypsinized with 0.05% Trypsin (Gibco) and a mix of Dispase (Stem Cell Technologies) and DNAse (Stem Cell Technologies). A portion of this cell suspension was stained with trypan blue and counted using the Countess Automated Cell Counter (Invitrogen). Based on the counting, the cells were diluted to the appropriate cell stock concentration for running on the 10X Chromium machine.

The cell suspensions were loaded on a 10X Genomics Chromium instrument to generate single-cell gel beads in emulsion (GEMs) for targeted retrieval of approximately 10,000 cells. Single-cell RNA-seq libraries were prepared using the following Single Cell 3’ Reagent Kits v3: Chromium™ Single Cell 3’ Library & Gel Bead Kit v3, PN-1000092; Single Cell 3’ Chip B Kit PN-1000074 and i7 Multiplex Kit PN-120262 (10X Genomics) and following the Single Cell 3’ Reagent Kits v3 User Guide (CG000183_ChromiumSingleCell3'_v3_UG_RevB). Libraries were run on an Illumina HiSeq 4000 as 2 × 150 paired-end reads. The Cell Ranger Single Cell Software Suite, version 3.1, was used to perform sample de-multiplexing, barcode and UMI processing, and single-cell 3′ gene counting. A detailed description of the pipeline and specific instructions to run it can be found at https://support.10X genomics.com/single-cell-gene-expression/software/pipelines/latest/installation. All generated fastqs were aligned to mouse (mm10) genome references contained within the Cell Ranger software.

### Availability of data and materials

The mixture of 3 cell lines and the FVB3-mammary, FVB4-mammary datasets are available from GEO: GSE136148 [27]. The Balbc-mammary dataset is published in this study (GEO: GSE165336) [63]. The TM 10X-mammary and TM Smartseq-mammary datasets were downloaded from the Tabula Muris Consortium website [57]. All the T cell datasets were downloaded from GEO (detailed GEO ID are provided in Additional file 2: Table S3) [32–35, 37, 64]. Note, only the 10X genomics data (from breast cancer patient samples 9, 10, and 11) in the Azizi et al. [35] paper were used in our study. In addition, the Oh et al. [37] bladder dataset has both treated and untreated cells, and we studied only the untreated cells to be consistent with the other datasets.

External validation datasets: The METABRIC dataset was obtained from the European Genome-Phenome Archive (accession number: EGAS00000000083) and clinical information from the original publication [39]. The Harrell 855 human breast tumor microarray data and the clinical data were downloaded from the Harrell et al. paper [40]. The TCGA BRCA gene expression data were downloaded from the Broad Institute TCGA GDAC Firehose (https://gdac.broadinstitute.org/), and the clinical data were downloaded from the Genomic Data Commons Data portal (https://portal.gdc.cancer.gov/projects/TCGA-BRCA). The clinical trial CALGB40601 gene expression data were downloaded from GEO: GSE116335 and dbGAP study accession phs001570.v2.p [65]. All datasets were normalized and log2 transformed prior to the gene signature analysis.

The MultiK software is implemented in R package and is freely available under the MIT license on Github: https://github.com/siyao-liu/MultiK [62], and deposited in Zenodo: 10.5281/zenodo.5138967 [66].

The Poisson modified synthetic data sets and the code to generate the simulated datasets have been deposited in Github: https://github.com/siyao-liu/MultiK [62] and Zenodo: 10.5281/zenodo.5138967 [66].

## Supplementary Information

**Additional file 1:.** Supplementary figures

**Additional file 2:.** Supplementary tables

**Additional file 3:.** Review history
