# Supplementary material for: MultiK: an automated tool to determine optimal cluster numbers in single-cell RNA sequencing data
Source: Genome Biol. 2021 Aug 19;22:232. doi: 10.1186/s13059-021-02445-5 (PMC8375188; doi:10.1186/s13059-021-02445-5)
Supplement: Supplementary file 1 — Additional file 1:. Supplementary figures [file 13059_2021_2445_MOESM1_ESM.docx]

Supplemental figures of “MultiK: an automated tool to determine optimal cluster numbers in single-cell RNA sequencing data”

This file includes all the figures that supplement the analysis in the main manuscript.

**Fig. S1. Heatmaps of low *K* and high *K* clusters in the 3 cell line mixture dataset.**

Fig. S1. Heatmaps of low *K* and high *K* clusters identified from MultiK (indicated by the colored bars and labels at the top) in the 3 cell line mixture dataset. (a) The top 20 differentially expressed genes at *K*=3 for each cluster are shown on the y-axis. (b) The top 10 differentially expressed genes at *K*=7 for each cluster are shown on the y-axis.

**Fig. S2. Synthetic experiment.**

Fig. S2. Synthetic experiment showed the sensitivity of MultiK in the identification of classes and subclasses. Each panel is a UMAP plot showing the synthetic experiment clusters identified by MultiK in the low-resolution space under each perturbation. The rows are the varying Poisson parameters: 1, 2, and 4. The columns are the varying numbers of modified genes: 20, 30, 50, 100, and 350. The colors represent the clusters identified by MultiK, and the text indicates whether the synthetic group is identified as a class, a subclass, or neither of the two. Fibro=Fibroblast, Pois=Poisson.

**Fig. S3. Robustness of MultiK in 3 simulated scRNA-seq.**


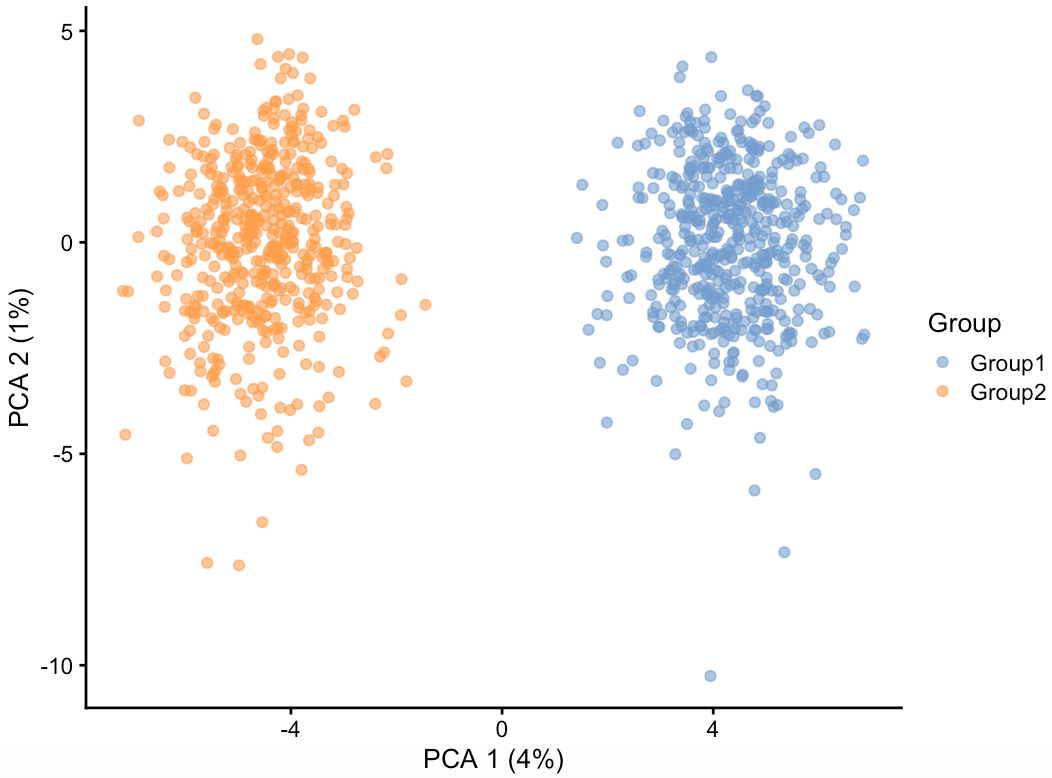

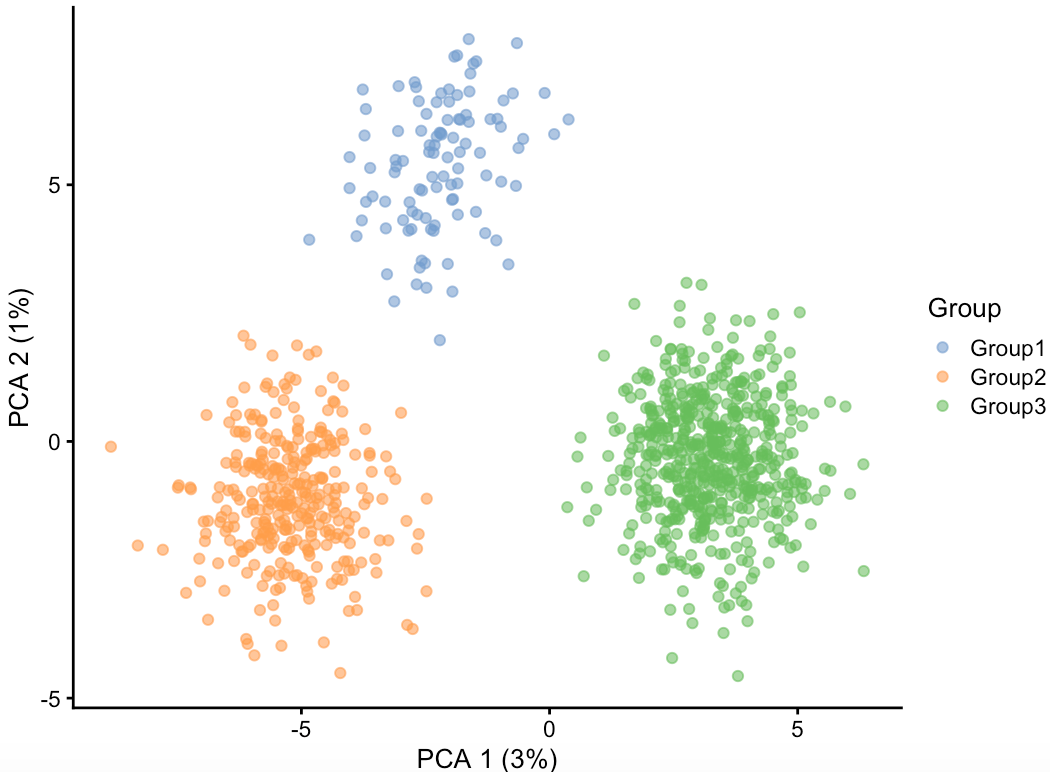

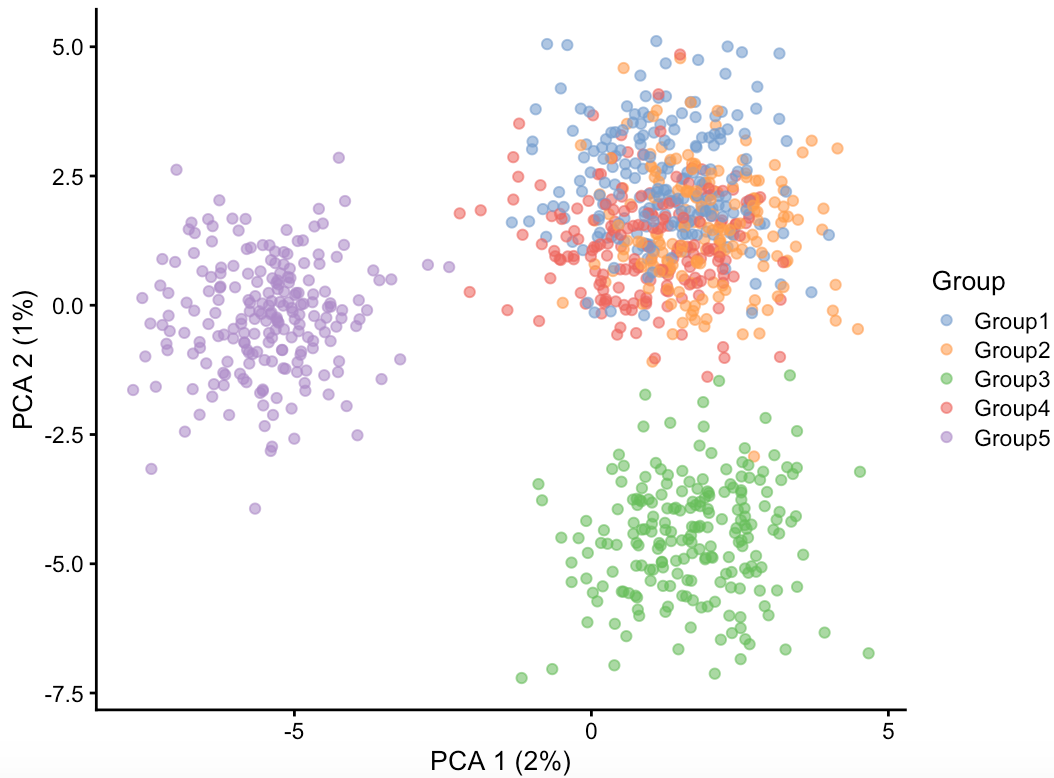


**a**

**b**

**c**

Fig. S3. Robustness of MultiK in identifying major cell groups in 3 simulated scRNA-seq datasets from R/Splatter package. Each dataset contained 5,000 genes and 1,000 cells. (a) Two equal groups; (b) Three unequal groups with probabilities of 0.1, 0.3, and 0.6; (c) Five equal groups. The rows in this figure are the 3 simulated datasets. The first column is the PCA scatterplot of the simulated data with PC1 scores on the X-axis and PC2 scores on the Y-axis. Each point is a cell, and the color indicates which group the cell comes from. The columns on the right are the MultiK diagnostic plots. Note that the diagnostic plots are missing in panel (c), because all the clustering runs give *K*=5.

**Fig. S4. Robustness of MultiK in detection of rare cell-types.**

**b**

**c**


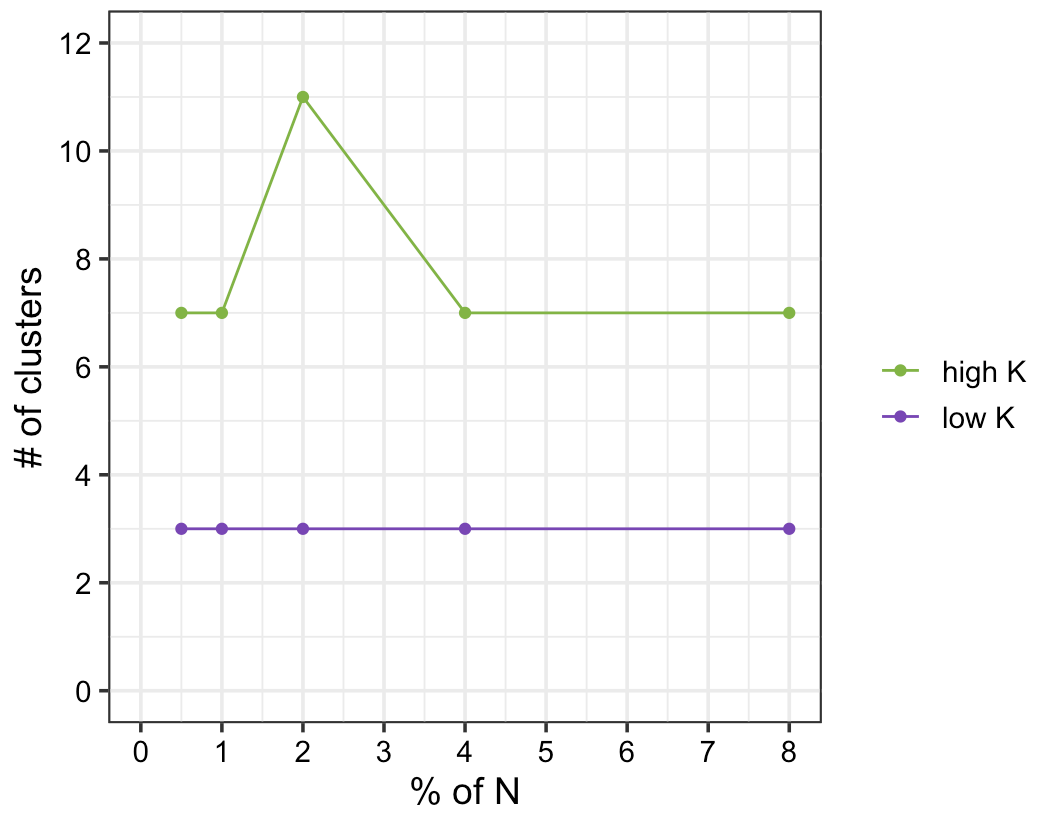


**a**

Fig. S4. Robustness of MultiK in detection of rare cell-types using the 3 cell line mixture dataset. Different percentages of the cells in the fibroblast (smallest) group of the 3 cell lines were removed. (a) The X-axis represents the percentage of fibroblast cells in the total number of cells in the dataset. The Y-axis represents the number of clusters suggested by MultiK. Low *K* solutions are plotted in purple whereas high *K* solutions are plotted in green. (b-c) MultiK diagnostic plots for the detection of rare cell-types in the 3 cell line mixture dataset. (b) 1% of the cells were fibroblast; (c) 2% of the cells were fibroblast. Left column is bar plot of the frequency of runs for each *K* across a total of 4,000 subsampling runs. The numbers above each vertical bar represent the frequency of runs. Dotted line is the frequency 100 threshold. Middle column is the plot of relative PAC score for each *K*. X-axis represents *K*. Y-axis represents the relative PAC score. Right column is the scatterplot of (1-rPAC) and the frequency of *K*. Each dot represents a level of *K*. The thin dotted line connects each *K* to show the ordering. The fat solid line connects the candidates for optimal *K*.

**Fig. S5. Heatmaps of low *K* and high *K* clusters in FVB3-mammary dataset.**

Fig. S5. Heatmaps of low *K* and high *K* clusters identified from MultiK (indicated by the colored bars and labels at the top) in FVB3-mammary dataset. The top 10 differentially expressed genes at *K*=8 (a) and *K*=18 (b) for each cluster are shown on the y-axis.

**Fig. S6. MultiK diagnostic plots for mouse mammary gland datasets.**

Fig. S6. MultiK diagnostic plots for FVB4-mammary, Balbc-mammary, TM 10X-mammary and TM Smartseq-mammary datasets. The first column is the bar plot of the number of clustering runs for each *K* across a total of 4,000 subsampling runs. The numbers above each vertical bar represents the actual number of runs. Dotted line is the 100 runs threshold. The second column is the plot of relative PAC score for each *K*. X-axis represents *K*. Y-axis represents the relative PAC score. The third column is the scatterplot of (1-rPAC) and the number of runs for each *K*. Each dot represents a level of *K*. The thin dotted line connects each *K* to show the ordering. The fat solid line connects the candidates for optimal *K*. (a-c) FVB4-mammary dataset, *K*=9, 18, 23 are identified as optimal. (d-f) Balbc-mammary dataset, *K*=10, 13, 23 are identified as optimal. (g-i) TM 10X-mammary dataset, *K*=15 is identified as optimal. (j-l) TM Smartseq-mammary dataset, *K*=7, 9, 10 are identified as optimal.

**Fig. S7. Heatmaps of GSA score across datasets.**

Fig. S7. Heatmaps of GSA score across datasets in low-resolution space and high-resolution space. (a,b) GSA score (> 0.75) in normal mouse mammary gland datasets.

Rows are gene sets defining each cluster in FVB3-mammary dataset. Columns are clusters identified from the other datasets. (c, d) GSA score (> 0.6) in human T cell datasets. Rows are gene sets defining each group in the Azizi et al. dataset. Columns are clusters identified from the other datasets. For datasets in which MultiK identified a single optimal *K* solution, the single optimal *K* was used in both low and high-resolution analysis. For datasets in which MultiK identified 3 optimal *K* solutions, the lowest *K* was used in the low-resolution analysis; if the second lowest *K* was more than 2 above the lowest *K*, then the second lowest *K* was used in the high-resolution analysis, otherwise the highest *K* was used in the high-resolution analysis. In the Zheng et al. dataset, *K*=18 and 19 were identified as optimal, but we chose *K*=6 in the low-resolution analysis, because the difference between *K*=18 and *K*=19 was very small, and *K*=6 seemed to be a good low *K* candidate after reviewing the MultiK diagnostic plot. TM= Tabula Muris, c=cluster, T=T cell cluster.

**Fig. S8. MultiK diagnostic plots for T cell datasets.**

Fig. S8. MultiK diagnostic plots for T cell datasets. The first column is the bar plot of the number of clustering runs for each *K* across a total of 4,000 subsampling runs. The numbers above each vertical bar represents the actual number of runs. Dotted line is the 100 runs threshold. The second column is the plot of relative PAC score for each *K*. X-axis represents *K*. Y-axis represents the relative PAC score. The third column is the scatterplot of (1-rPAC) and the number of runs for each *K*. Each dot represents a level of *K*. The thin dotted line connects each *K* to show the ordering. The fat solid line connects the candidates for optimal *K*. (a-c) Savas et al. dataset, *K*=4, 15 are identified as optimal. (d-f) Guo et al. dataset, *K*=18, 19, 22 are identified as optimal. (g-i) Zheng et al. dataset, *K*=18, 19 are identified as optimal. (j-l) Zhang et al. dataset, *K*=6, 15, 23 are identified as optimal. (m-o) Oh et al. dataset, *K*=5 is identified as optimal. (p-r) Azizi et al. dataset, *K*=13, 27 are identified as optimal.

**Fig. S9. Reproducible T cell groups derived from high-resolution space across datasets.**

Fig. S9. Reproducible T cell groups derived from high-resolution space across datasets using Azizi et al. as reference. (a) 2-d UMAP plot of Louvain clustering from Seurat (version 3.1.2) on the full data matrix at *K*=27. Each dot represents a single cell and each color present a cluster at a given *K* level. (b) Heatmap of reproducible T cell groups found in the other dataset using gene set enrichment analysis. Left is a dendrogram constructed from the cluster means in the Azizi et al. dataset. Closed circles on the nodes indicate significant SigClust *p* value (< 0.05) suggesting a cluster is class, whereas open circles on the nodes indicate non-significant SigClust *p* value (≥ 0.05) suggesting a cluster is subclass. Right is a heatmap showing the presence (colored black) or absence (colored grey) of a group identified from the Azizi et al. dataset in the other datasets. The reproducible groups are highlighted with asterisks at the end of each row in the heatmap.

**Fig. S10. Prognostic effect of reproducible T cell gene signatures.**

**a**

**b**

**c**

**d**

Fig. S10. Prognostic effect of reproducible T cell gene signatures compared to other T cell signatures as well as single gene expression (indicated on the y-axis, see Methods) as continuous variables in external breast cancer datasets (Panel a shows all patients in each individual dataset, panels b-d show stratified clinical groups in each individual dataset). Forest plots show hazard ratio (circles) and 95% confidence intervals (horizontal ranges) derived from the Cox proportional hazards model for overall survival in univariate analysis. Red indicates significant hazard ratio estimates (*p* value < 0.05) whereas blue indicates non-significant estimates (*p* value ≥ 0.05).
