# Supplementary material for: MultiK: an automated tool to determine optimal cluster numbers in single-cell RNA sequencing data
Source: Genome Biol. 2021 Aug 19;22:232. doi: 10.1186/s13059-021-02445-5 (PMC8375188; doi:10.1186/s13059-021-02445-5)
Supplement: Supplementary file 3 — Additional file 3:. Review history [file 13059_2021_2445_MOESM3_ESM.docx]

Review History

**First round of review**

**Reviewer 1**

**Were you able to assess all statistics in the manuscript, including the appropriateness of statistical tests used?**

No

**Were you able to directly test the methods?**

No

**Comments to author:**

Estimating the optimal number of data-driven clusters is a critical issue in unsupervised clustering analysis. Existing tools often choose a K subjectively, with interpretations largely relying on users' preferences (e.g., desired clustering resolution) and/or prior knowledge. To solve this problem, the authors developed MultiK to auto-select the optimal number of cell clusters via a multi-resolution and consensus manner. MultiK achieved better performance on synthetic datasets than Seurat, SC3, and IKAP, and was proved to be robust and capable for large scRNA-seq dataset analysis. The further applications on mouse mammary gland and human T cell datasets showed that the hierarchy cell cluster structure fit the stratified biological cell groups well, based on differential gene expression and pathway enrichment analysis. Overall, the method is well-described with rigorous details. However, some issues still exist and more insights are needed regarding the result interpretation and conclusion making. More details are listed below.

Major:

*	Considering diverse and abundant scRNA-seq data in the public domain, (i) will the performance of MultiK be affected by different cell numbers and read depth? If yes, how? (2) will different sequencing technologies (e.g., 10X genomics and Smart-Seq) have impacts on the result or show any tendencies in MultiK?

*	Will gene imputation in the preprocess step affect (enhance or fail or no significant effect) the result of MultiK results? Is there a preference whether gene imputation should or should not be used, and why?

*	Fundamentally, MultiK is designed for cell clustering, no matter the auto K selection or hierarchical structure it can deliver. Only compare MultiK with Seurat, SC3, and IKAP is not sufficient. The author should add experiments to compare MultiK with more tools, such as BackSPIN, TooManyCells, and scGNN.

*	When comparing the clustering results of MultiK with other tools. Multiple indices were mentioned in the introduction section, such as the Silhouette index, GAP-statistic, Clest, and prediction strength, yet not been used in the evaluations. The description of the benchmark in Line 179-186 did not include any statistic scores, making the benchmarking part weak and unreproducible. Also, the Seurat result was not even mentioned in the content.

*	The authors keep highlighting that MultiK can provide biological insights/relevance to subclasses, however, the evaluation process did not seem to include any biological steps, and it is unclear what the insight into biology will be. Specifically, rather than only using differentially expressed genes and pathway enrichment tests, the author should also look into TF-gene regulations in a biological system to show the true biological insights. Also, a case that can link cell classes and subclasses with cell lineage or developmental trajectory would be very nice.

*	The author claimed in the cover letter that the MultiK code is submitted as a supplementary file, but I could not find it in the system. I could not make any comments on the package/tutorial of this tool. I will suggest uploading the package on GitHub and claim it in the code availability section.

Minor:

*	Seurat clustering should be Louvain clustering

*	Appropriate references are needed to justify the rationale of data synthesis.

*	I am not sure what is the necessity to describe the details of the experimental design of Balbc data. Is it the data newly generated?

*	What is the threshold for DEG determination?

**Reviewer 2**

**Were you able to assess all statistics in the manuscript, including the appropriateness of statistical tests used?**

Yes: Most of the statistical methods are detailed described in the manuscript. However, I could not access the code and thus unable to test the tool, check the implementation or the reproducibility.

**Were you able to directly test the methods?**

No

**Comments to author:**

Liu et al. describe a tool, named "MultiK", which uses a consensus approach to derive optimal number of groups K in single cell RNA-seq cluster analysis. MultiK first makes 4000 Seurat runs with cell subsamples and range of resolution parameters. Cluster number frequency and rPAC scores are further calculated and plotted. Optimal number of clusters K are two values that "maximizes frequency and minimizes rPAC" (i.e. top right corner). At the end, MultiK builds a dendrogram of cluster centroids with hierarchical clustering and determines cluster split significance with SigClust. Validation of MultiK and comparison with other methods are performed with a mix cell line data set and a synthetic (in silico spike-in) experiment. The manuscript further shows applications of MultiK on two biological data sets.

Overall the authors have mostly thoroughly explained their approach and model. The model is quite important in scRNA-seq analysis as clustering is an essential step in the general pipeline. However, please consider the following comments and questions.

Major

1. The authors design a synthetic experiment to estimate the sensitivity and specificity of MultiK in comparison to Seurat, SC3 and IKAP. I understand the author's intention to create a cell subset. However, I have a few questions and comments

a. A simple Poisson distribution is quite different from the real single cell gene expression distribution (dropout, expression bursting). Would it be better if we add or subtract Poisson noise as signals rather than overwrite the expression?

b. Will alternating different subsets of synthetic genes affects sensitivity?

c. Since we know the true cell label, a plot of cluster accuracy metrics (such as ARI, Precision and Recall) across methods of comparison could help illustrate the improvements and limitations of MultiK.

2. In the application example, I am curious to know how well did the other methods perform? This will help illustrate the performance of MultiK.

3. The author briefly mentioned decisions on some key parameters, including subsampling percentage, number of PCs, number of vst genes, and number of nearest neighbors. How robust / stable is MultiK regarding changes of these parameters? Is it possible to add some visualization or tables to prove the point?

4. I was not able to locate the code for this manuscript. Please include it for the review. In addition, an easy to install package, Github repository or Bioconductor implementation with examples can help distribute the package and benefit the community.

5. Computational intensity is the main concern for me for MultiK. I wonder if we could compare the CPU time (or User time) across different methods? Is multi-core / parallel implementation available for MultiK? How much RAM do we need to run such an analysis given a number of cells? Any guideline would help the users' decision on analysis package selection.

Minor

1. In the GSA analysis, the author only includes upregulated genes. Any reason for that?

2. Split Figure 1 into sub panel a and b can help clean the references in the manuscript.

3. The header in Supplement Table 2 seems to be shifted and causes mismatch. Please fix

4. The author mentioned that splits between B cells and T cells are not significant in the FVB3-mammary example. Is this due to the small number of these cells? Is it possible to try with a smaller near neighbor parameter in Seurat (higher resolution) and see if they will be recovered?

5. In Figure 3e, the Fibro5 was labeled Neural in 3d. Is Firbo5 mislabeled? Or is Fibro5 more similar to Neural?

6. In Figure S5, a and b reordered the clusters (x axis) which helped a lot with the visualization. Can we do the same reordering for subplot c and d?

**Authors Response**

**Point-by-point responses to the reviewers’ comments:**

*We thank the reviewers for their helpful feedback and suggestions, and we have incorporated a number of new analyses in the manuscript as suggested by the reviewers. We do wish to note, however, that some of the reviewers’ comments were focused on the details of clustering algorithms and clustering methods. We feel that our work here is not about clustering methods, and that the main point of MultiK is to determine the optimal number(s) of clusters. In fact, our MultiK method does not propose a novel clustering algorithm, and instead is based on an established and widely-used clustering method (the Louvain clustering implemented in Seurat), and thus our paper is not about optimizing and testing various means of improving clustering methods. We appreciate the reviewers’ concern on how different cell numbers, read depth, and other key parameters such as number of PCs, number of selected features, etc., affect the performance of MultiK. These are indeed critical issues in clustering, and each of these parameters would surely impact the clustering results. However, the main focus of our paper is not to tune every clustering parameter, but instead was to develop a method that can objectively assess the number of clusters/groups present. That is why we keep many of these clustering parameters the same as the default parameter settings in many of the established methods. We did explore some of these clustering parameters and looked at how varying these parameters impacts the performance of MultiK, and the detailed results can be found in the point by point responses below in blue text.*

Point-by-point responses to the reviewers’ comments:

Responses to Reviewer #1:

Reviewer #1: Estimating the optimal number of data-driven clusters is a critical issue in unsupervised clustering analysis. Existing tools often choose a K subjectively, with interpretations largely relying on users' preferences (e.g., desired clustering resolution) and/or prior knowledge. To solve this problem, the authors developed MultiK to auto-select the optimal number of cell clusters via a multi-resolution and consensus manner. MultiK achieved better performance on synthetic datasets than Seurat, SC3, and IKAP, and was proved to be robust and capable for large scRNA-seq dataset analysis. The further applications on mouse mammary gland and human T cell datasets showed that the hierarchy cell cluster structure fit the stratified biological cell groups well, based on differential gene expression and pathway enrichment analysis. Overall, the method is well-described with rigorous details. However, some issues still exist and more insights are needed regarding the results, interpretation and conclusion making. More details are listed below.

Major:

* Considering diverse and abundant scRNA-seq data in the public domain, (i) will the performance of MultiK be affected by different cell numbers and read depth? If yes, how?

*Response: MultiK will be affected by these features because it is based on clustering methods, and clustering methods performance can be impacted by different cell numbers and different read depths. We have addressed this issue by focusing on the cell number question, and provided a table (Rebuttal Letter Supplementary Table 1) comparing how different cell numbers affect the number of optimal clusters identified by MultiK using the 3 cell line mixture dataset. As the cell number increases, the number of optimal clusters in the low K space is either 2 or 3 (3 being the true number), and the number of optimal clusters in the high K space has larger effects. It is expected that when the cell number is low, for example, N=150 cells, the smallest group (fibroblast ~10% of cells) may be missed, because there are too few cells to be detected. This analysis suggests around 37 fibroblast cells (N=450 total cells) are sufficient for MultiK to detect this most rare group as a cluster/group in the high K space, and ~50 cells (N=500 total cells) in the low K space.*

*For read depth, we would like to note that the datasets used in our application examples answered this question to some extent. In Table S3, we showed detailed information about each dataset including the sequencing technology. We included datasets generated from both 10X genomics and Smart-seq2 platforms, where the former is known to have lower read depth coverage but higher cell number, and vice versa for the latter. We did not see major differences between the two technologies in terms of the MultiK performance. Having said that, we agree with the reviewer that understanding how different read depths would be an interesting topic itself to study further in the future, but this is more related to a clustering parameter than a MultiK tuning parameter, and thus not further pursued here.*

(2) will different sequencing technologies (e.g., 10X genomics and Smart-Seq) have impacts on the result or show any tendencies in MultiK?

*Response: As discussed above, in our real dataset application (both mouse mammary gland and human T cell datasets), three of which are Smart-seq2 and the rest are 10X genomics (see Table S3), we did not see major differences from the sequencing technology/platform in the results of MultiK. We say this because we found the same reproducible groups across both platforms in our presented studies. Specifically, in the mouse mammary gland case, we found 6/7 reproducible groups on both 10X and Smart-seq2 platforms in the low K space, and all 8 reproducible groups on both platforms in the high K space (Figure 4, Supplementary Table 16). In the human T cell case, all the reproducible groups were detected by both platforms in both low K and high K space (Figure 5, Figure S7, Supplementary Tables 15, 17). This indicates that our MultiK method is robust to different single cell sequencing technologies.*

* Will gene imputation in the preprocess step affect (enhance or fail or no significant effect) the result of MultiK results? Is there a preference whether gene imputation should or should not be used, and why?

*Response: We believe gene imputation in the preprocessing step will affect MultiK results, however, this is much more of a clustering aspect than a MultiK performance aspect. We note that there are a good number of gene imputation methods developed for scRNAseq data such as MAGIC[1], SAVER[2], and scGNN[3] (which is a novel method that combines gene imputation and clustering using a graph neural network framework). However, evaluating different gene imputation methods is beyond the scope of this paper, and is truly a paper in and of itself. In fact, some studies have addressed this explicitly and for example, Hou et al.[4] performed a systematic evaluation of 18 scRNAseq imputation methods, and concluded that most of the imputation methods failed to improve clustering and trajectory analysis compared to applying no imputation. In addition, most of the imputation methods employ the information from the dataset itself rather than external information, which biases the imputation results[5]. Lastly, the goal of MultiK is to provide objective guidance on the estimation of K. For these reasons, we have chosen not to study gene imputation here but do acknowledge that to impute or not is a very important clustering question.*

* Fundamentally, MultiK is designed for cell clustering, no matter the auto K selection or hierarchical structure it can deliver. Only compare MultiK with Seurat, SC3, and IKAP is not sufficient. The author should add experiments to compare MultiK with more tools, such as BackSPIN, TooManyCells, and scGNN.

*Response: We appreciate the reviewer’s suggestion on comparing the performance of MultiK with additional similar methods. First, we would like to point out that MultiK is not a new clustering method, but rather a method that uses an established clustering method (Louvain clustering in Seurat), and its goal is to automatically selects K when given a single data set; the automatic selection of K is the novel contribution. That is why we focused on comparing MultiK to IKAP and SC3, as these methods are designed for determining K in scRNAseq data. We also decided to compare MultiK to the Seurat default resolution parameter, because we wanted to see how MultiK (which considers multiple resolution parameters) performs vs. a single default setting resolution parameter in a widely used package.*

*Second, it is worth noting that the tools suggested by the reviewer here do not provide an objective assessment of what is the data driven optimal K. BackSPIN, TooManyCells, and scGNN were designed for clustering scRNAseq data, especially in the important and challenging case of identifying rare cell populations. BackSPIN uses a divisive biclustering algorithm to identify clusters, and iteratively applies feature selection and cell clustering, which thus may have a tendency to over-partition the data[6]. scGNN uses a graph neural network framework and combines gene imputation and cell clustering, but essentially it is developed for clustering cells. While TooManyCells employs a multi-resolution idea, and provides a tree structure visualization to study the relationship between cell clades, it does not provide an objective determination as to how many clades are statistically significant; instead TooManyCells leaves to the viewer to determine how many groups they feel are present. Because these methods cannot provide an objective assessment of K, it is not possible for us to compare MultiK to these methods. If we do these comparisons, we would have to either rely on the default clustering resolution and/or make a subjective decision on what K to use from these 3 methods, and make a subjective decision about what is the optimal K, which would not provide a fair comparison. This was also exactly why we developed “MultiK”, a tool that provides the user, using the data in hand, a data driven number of "clusters" or "groups"; conversely many clustering tools provide a visualization tool to explore clusters/groups at different resolutions, but then leaves it to the scientist to literally eyeball how many "groups" they think are present.*

*We also now provide more discussion regarding this key point in the Discussion section in the revised manuscript.*

* When comparing the clustering results of MultiK with other tools. Multiple indices were mentioned in the introduction section, such as the Silhouette index, GAP-statistic, Clest, and prediction strength, yet not been used in the evaluations. The description of the benchmark in Line 179-186 did not include any statistic scores, making the benchmarking part weak and unreproducible. Also, the Seurat result was not even mentioned in the content.

*Response: We appreciate the reviewer’s comment regarding the evaluation metrics in the benchmarking analysis. First, we would like to point out that MultiK is not about clustering, but rather a tool that provides guidance on how to choose useful values of K. Next, we used SigClust analysis implemented in MultiK as an objective statistical assessment of the clusters/groups identified by MultiK. SigClust performs a hypothesis test for assessing the significance of the clustering result defined groups[7,8]. All the clusters identified by MultiK at the optimal K levels are assessed by SigClust, and thus each group is statistically tested to determine if it is truly unique (i.e. a class) or is less distinct (i.e. a subclass). Second, we would like to note that IKAP does not provide individual cell labels at the optimal K level, and therefore we cannot perform statistics on the clusters identified from IKAP. For Seurat, we used the default resolution parameter to compare with the optimal K(s) from MultiK, which utilizes multiple resolution parameters, but the Seurat default does not provide an optimal K, and thus it does not seem to make sense to perform statistics on the clusters that are at a default setting, which can be easily changed; again this is the very point that motivated us to develop MultiK.*

*For SC3, it does provide the optimal K estimation, but to obtain the cell labels at the estimated K level, one needs to run the clustering function providing the estimated K as an input. We have now updated Supplemental Table 2 to reflect more details in the benchmarking results using the synthetic experiments. We observed that the Seurat default setting detected the synthetic group in all the synthetic cases, and agreed with the high K solution in MultiK. This was not unexpected, because MultiK uses a multi-resolution approach for choosing K. On the other hand, SC3 estimated far more clusters in all the cases compared to MultiK, and was more sensitive in detecting the synthetic group. However, the detection in SC3 seemed to be unstable, as sometimes SC3 detected all the modified cells as a synthetic group and sometimes it broke the modified cells into 2 groups. IKAP generally agreed with the low K solution in MultiK. However, since IKAP does not provide cell labels at the best K, we used the UMAP plots generated by IKAP at the best K, and eyeballed whether the synthetic group was detected or not (last column in Supplemental Table 2). We observed that IKAP was not as sensitive as MultiK in detecting the synthetic group, for example, at Poisson parameter 1, only 100 modified genes were needed for MultiK to detect the synthetic group as a subclass, but it required 350 genes for IKAP to detect the synthetic group. Lastly, the Seurat result in the benchmarking analysis was mentioned in line 181 in the old version of the manuscript.*

* The authors keep highlighting that MultiK can provide biological insights/relevance to subclasses, however, the evaluation process did not seem to include any biological steps, and it is unclear what the insight into biology will be. Specifically, rather than only using differentially expressed genes and pathway enrichment tests, the author should also look into TF-gene regulations in a biological system to show the true biological insights. Also, a case that can link cell classes and subclasses with cell lineage or developmental trajectory would be very nice.

*Response: We appreciate the reviewer’s suggestion on looking into transcription factor regulation and trajectory analysis, however, for the reproducible groups identified in the mouse mammary gland datasets, the basal, luminal, endothelial, fibroblasts, and immune groups are already well known biologically and have been well characterized and studied in the literature extensively[9–12]. Thus, for the mouse mammary gland data our ‘found’ groups are biological validated groups, representing distinct cell lineages, and hence these results are biologically informative and provide validation of the findings of our novel method. Hence, we feel there is no need for additional bioinformatics transcription factor analysis to show that the discovered groups are biologically valid.*

*For the reproducible groups found in the T cell datasets, many of them are known and previously validated to be biologically important, and we point out a number of previously defined T cell subset ‘marker’ genes, and thus, these finding of previously defined marker genes in our newly defined groups is biological validation of our groups. In addition, to provide even higher-level biological validation, we performed survival analysis to show that these reproducible T cell groups have prognostic value, and in fact many were highly prognostic. For example, the rediscovery of the resident memory T cell group (originally discovered in Savas et al.[13]), showed the importance of the biology of this T cell group. In summary, we strongly feel our ‘reidentification’ of known groups in the mouse mammary gland data, and the identification of multiple T cell groups with strong survival prediction abilities and expression of previously defined T cell subset marker genes, provides the biological validation of our MultiK discovered groups.*

* The author claimed in the cover letter that the MultiK code is submitted as a supplementary file, but I could not find it in the system. I could not make any comments on the package/tutorial of this tool. I will suggest uploading the package on GitHub and claim it in the code availability section.

*Response: We apologize for this lack of the code, and we note we had significant difficulties loading the code into the manuscript system. The MultiK code has now been deposited in Github: https://github.com/siyao-liu/MultiK and is publicly available. A short tutorial along with an example is also provided.*

Minor:

* Seurat clustering should be Louvain clustering

*Response: Agreed. We have now clarified this throughout the entire manuscript.*

* Appropriate references are needed to justify the rationale of data synthesis.

*Response: The reasons for generating the synthetic datasets from the 3 cell line mixture dataset using our methods are the following:*

*It is still very difficult to simulate scRNAseq data that mimics real-world data. Thus, we chose to avoid using simulation packages to generate entire scRNAseq data, because the underlying model is still difficult and requires further investigation. Instead, we took real world data and systematically modified just a small part of this, thus leaving the real-world data structure largely untouched.*

*We already have a 3 cell line mixture dataset, in which the cell identities are known, providing a ground truth dataset for us to compare the performance of different methods. A simple way to augment the data is to introduce a synthetic cluster/group of cells by adding in some signals to the original data.*

*We chose to modify the gene counts by fitting a Poisson distribution with various Poisson parameters, and this allowed us to see the impact of the signal that we purposely added in the data. It also allowed us to identify the contexts where the synthetic cluster can be identified as a class or a subclass. The reason why we chose to use a Poisson distribution is that multiple recent studies have shown that UMI count scRNAseq data is not significantly zero-inflated and any observed zero-inflation is likely driven by the cell-type heterogeneity and biological variation [14,15]. Particularly, Kim et al.[15] observed that the majority of the genes fit the expected Poisson curve in a homogeneous cell population, and their results strongly suggested that it is unnecessary to model any zero counts using a zero-inflated distribution. Inspired by that, we think it is appropriate to use a simple Poisson distribution to modify a small subset of genes in a subset of luminal cells in our 3 cell line mixture dataset.*

*We acknowledge that there could be many other ways to generate synthetic scRNAseq data, but this is not the focus of this paper. Our goal here was to generate a set of synthetic datasets with ground truth labels and use them for benchmarking the estimation of cluster number provided by MultiK. Note the synthetic data was just one of multiple ways we benchmarked our method.*

* I am not sure what is the necessity to describe the details of the experimental design of Balbc data. Is it the data newly generated?

*Response: Yes, the Balbc scRNA-seq data is new to this paper and thus required an experimental description.*

* What is the threshold for DEG determination?

*Response: For DEG determination, we did group 1 vs. all others, group 2 vs. all others, etc. using the Wilcoxon rank sum test. We used a Bonferroni-corrected p value ≤ 0.05 as the cutoff to identify significant DEGs. Details can be found in the Methods/Differential expression analysis in the main manuscript.*

Responses to Reviewer #2:

Reviewer #2: Liu et al. describe a tool, named "MultiK", which uses a consensus approach to derive optimal number of groups K in single cell RNA-seq cluster analysis. MultiK first makes 4000 Seurat runs with cell subsamples and range of resolution parameters. Cluster number frequency and rPAC scores are further calculated and plotted. Optimal number of clusters K are two values that "maximizes frequency and minimizes rPAC" (i.e. top right corner). At the end, MultiK builds a dendrogram of cluster centroids with hierarchical clustering and determines cluster split significance with SigClust. Validation of MultiK and comparison with other methods are performed with a mix cell line data set and a synthetic (in silico spike-in) experiment. The manuscript further shows applications of MultiK on two biological data sets.

Overall the authors have mostly thoroughly explained their approach and model. The model is quite important in scRNA-seq analysis as clustering is an essential step in the general pipeline. However, please consider the following comments and questions.

Major

1. The authors design a synthetic experiment to estimate the sensitivity and specificity of MultiK in comparison to Seurat, SC3 and IKAP. I understand the author's intention to create a cell subset. However, I have a few questions and comments

a. A simple Poisson distribution is quite different from the real single cell gene expression distribution (dropout, expression bursting). Would it be better if we add or subtract Poisson noise as signals rather than overwrite the expression?

*Response: We agree with the reviewer that due to dropout events and expression bursting, it is quite difficult to generate synthetic scRNAseq data that mimic real-world scRNAseq data. However, we do not think changing replacement to adding and subtraction would give us substantial different results than what we have already obtained. This is because most counts in scRNAseq data are naturally zeros.*

*As noted above, we chose to modify the gene counts by fitting a Poisson distribution with various Poisson parameters, and this allowed us to see the impact of the signal that we purposely added into the data. It also allowed us to identify the contexts where the synthetic cluster can be identified as a class or a subclass. Again, as noted above, the reason why we chose to use a Poisson distribution is that multiple recent studies have shown that UMI count scRNAseq data is not zero-inflated and the zero-inflation is likely driven by the cell-type heterogeneity and biological variation [14,15]. Particularly, Kim et al.[15] observed that the majority of the genes fit the expected Poisson curve in a homogeneous cell population, and their results strongly suggested that it is unnecessary to model the excessive zero counts using a zero-inflated distribution. Inspired by that, we think it is appropriate to use a simple Poisson distribution to modify a subset of genes in a subset of luminal cells in our 3 cell line mixture dataset. Lastly, we again point out that the synthetic data was just 1 of 4 means we use to assess the performance of MultiK, and that it is beyond the scope of this paper to be evaluating the best method(s) for generating novel synthetic data.*

b. Will alternating different subsets of synthetic genes affects sensitivity?

*Response: Yes, we suspect that alternating different subsets of synthetic genes will have some small impact on the sensitivity performance of the method. However, we viewed it more important to study the impact of the number of the synthetic genes as opposed to which set of genes to augment with synthetic signals. Hence, we varied the number of genes in the random set to focus on how different numbers of synthetic genes affect the sensitivity and identify contexts where MultiK is able to identify the synthetic group as a class or subclass.*

c. Since we know the true cell label, a plot of cluster accuracy metrics (such as ARI, Precision and Recall) across methods of comparison could help illustrate the improvements and limitations of MultiK.

*Response: First, as noted above, MultiK is not a novel clustering method, but rather a tool that provides guidance on how to choose a useful K. Calculating ARI or Precision and Recall are useful for comparing clustering algorithms. We developed MultiK to objectively assess K in the data. In addition, as noted in the response to Reviewer #1, IKAP does not provide cell labels at the optimal K level, and therefore we cannot calculate such statistics on the clusters identified by IKAP. For Seurat, we used the default resolution parameter to compare with the optimal K(s) from MultiK, which utilizes multiple resolution parameters, but the Seurat default does not aim to provide an optimal K, thus it does not seem to make much sense to calculate such statistics on the clusters that are not at the optimal K level. Second, the SigClust analysis implemented in MultiK is a valid statistical assessment of the clusters identified by MultiK. SigClust performs a hypothesis test for assessing the significance of the clustering results[7,8]. All the clusters identified by MultiK at the optimal K levels are assessed by SigClust.*

*Also, as noted in the response to Reviewer #1, we have now updated the Supplemental Table 2 to reflect more details in the benchmarking results using the synthetic experiments. We observed that the Seurat default setting detected the synthetic group in all the synthetic cases, and more or less agreed with the high K solution in MultiK. This was not unexpected, because MultiK uses a multi-resolution approach for choosing K. On the other hand, SC3 estimated far more clusters in all the cases compared to MultiK, and was more sensitive in detecting the synthetic group. However, the detection in SC3 seemed to be unstable, as sometimes SC3 detected all the modified cells as a syntenic group and sometimes it broke the modified cells into 2 groups. IKAP generally agreed with the low K solution in MultiK. However, since IKAP does not provide cell labels at the best K, we used the UMAP plots generated by IKAP at the best K, and eyeballed whether the synthetic group was detected or not (last column in Supplemental Table 2). We observed that IKAP was not as sensitive as MultiK in detecting the synthetic group, for example, at Poisson parameter 1, only 100 modified genes were needed for MultiK to detect the synthetic group as a subclass, but it required 350 genes for IKAP to detect the synthetic group.*

2. In the application example, I am curious to know how well did the other methods perform? This will help illustrate the performance of MultiK.

*Response: We have chosen not to benchmark MultiK against other methods in the application datasets because of the difficulty in establishing a ground truth to benchmark against. For all the real-world datasets except the 3 cell line mixture, the true cell labels are not known, and it would be unfair to use the cell labels that were identified computationally from one method as the ground truth to then compare versus another method. That’s why we chose to use the 3 cell line mixture dataset, and the synthetic modified version of the 3 cell line dataset, to perform the benchmark analysis; this is the only dataset tested where the ground truth is known, and here we did compare MultiK to other methods.*

3. The author briefly mentioned decisions on some key parameters, including subsampling percentage, number of PCs, number of vst genes, and number of nearest neighbors. How robust / stable is MultiK regarding changes of these parameters? Is it possible to add some visualization or tables to prove the point?

*Response: We appreciate the reviewer’s comments on the choice of the key parameters in MultiK. First, we would like to note that these are important clustering parameters and certainly more work is needed to evaluate how these key parameters impact clustering. However, our MultiK method is not about clustering, but instead provides objective guidance on how to choose optimal K(s) in the data. The goal of MultiK is not to optimize every possible clustering parameter for best clustering performance either, that in itself would be a paper. However, we did some exploration on a few of the key clustering parameters mentioned above (see Tables below); however, because the information content seems to be low, we decided not to include these results in the paper but do provide these results here.*

*Namely, the analysis of impact of principal component (PC) and the resolution parameter range is given in Rebuttal Letter Supplementary Table 2 and 3, and the analysis of the impact of k-nearest neighbor (KNN) is given in Rebuttal Letter Supplementary Table 4. Our conclusion from this analysis is that, at a given # of PCs, MultiK is very robust to the resolution parameter range (Table 2, Table 3), and thus we chose to the resolution parameter range 0.05 to 2.0 with increment of 0.05 in the MultiK algorithm. At a given resolution parameter range, the high K space solution in MultiK is not very stable in the 3 cell line mixture dataset when PC number is low (Table 2), but it seems to stabilizes after 30 PCs. In the FVB3-mammary dataset, the high K solution stabilizes after 30 PCs, but the low K solution has some fluctuation across all the PCs compared. Thus, we used 30 PCs for our default parameter in MultiK.*

*For the impact of KNN parameter in the Louvain clustering (k), we observed that the MultiK solutions are generally stable in both datasets being tested. We chose to use 20 nearest neighbors as the default setting in MultiK, because 20 is the default in Seurat. However, this k-nearest neighbor parameter indeed is an interesting clustering parameter to study further in the future.*

*For the consensus clustering subsampling percentage parameter, the standard setting in the field is 80% or 90% resampling (i.e. 20% or 10% withheld each time), and we took the default setting 80% from the established method called Consensus Cluster Plus[16], which was a tool that objectively assesses the number of groups from bulk gene expression data.*

*For the number of vst genes in the Louvain clustering, 2,000 is a community-wide standard on the number of genes to use for the downstream analysis. We did not explore here the impact of varying the number of vst genes as we felt this really a main point of clustering methods, and thus beyond the scope of our work here.*

4. I was not able to locate the code for this manuscript. Please include it for the review. In addition, an easy to install package, Github repository or Bioconductor implementation with examples can help distribute the package and benefit the community.

*Response: We again apologize for not providing the code. The MultiK code has been deposited in Github: https://github.com/siyao-liu/MultiK, and a short tutorial along with an example has also been provided.*

5. Computational intensity is the main concern for me for MultiK. I wonder if we could compare the CPU time (or User time) across different methods? Is multi-core / parallel implementation available for MultiK? How much RAM do we need to run such an analysis given a number of cells? Any guideline would help the users' decision on analysis package selection.

*Response: We appreciate the reviewer’s concern about the computational speed and admit this is one of the weaknesses of MultiK. This is indeed a limitation of using any subsampling approach. In response to this, we have carried out a timing experiment comparing the computational speed of MultiK and the other methods. Specifically, we randomly subsampled 1k, 2k, 5k, 10k, and 20k cells from the Azizi et al. dataset (BC9-11, N=~27k cells), and recorded the user time (in seconds) of all methods (MultiK, SC3, and IKAP) on the University of North Carolina Longleaf compute cluster (single CPU, 1 core, 500 GB RAM). We provide a table (Rebuttal Letter Supplementary Table 5) showing the results. Among all three methods, when the cell number is small (<2k), IKAP is the fastest, followed by MultiK, but the computation time increases dramatically as the cell number increases for both IKAP and MultiK. For example, from 1k to 2k, IKAP takes ~2 times longer; from 2k to 5k, IKAP takes ~7 times longer. Also, it is important to point out that IKAP does not provide cell labels at the optimal K level. When the cell number is large (>2k), SC3 is the fastest, and the computing time for SC3 does not seem to depend on the number of cells. However, it is worth noting that we are only considering the estimating K step in SC3 here. Users need to run extra clustering analysis in SC3 in order to obtain the cluster labels at estimated K.*

*Despite the longer computing times MultiK takes compared to other methods, the subsampling approach that MultiK implements is still an extremely rigorous way of finding robust clusters/groups. Improving the computation speed is an interesting computer science problem, but we do feel that the times needed to run MultiK are not prohibitive, and that these times will shorten as we and others works on means of optimizing the algorithm and implementation methods.*

Minor

1. In the GSA analysis, the author only includes upregulated genes. Any reason for that?

*Response: The reasons why we chose to use the positive/upregulated genes to trace a cluster/group is that genes with low/no expression gives a less definitive notion of cluster identity, basically the genes whose high expression define a cluster/group are unambiguously present in that group and tell us features that are present in a given group. In particular, when a cluster has “low expression of genes”, it is not clear whether the gene was never expressed, or its expression goes from high to low, etc. Focusing on genes with high expression to define a group causes much less ambiguity in terms of interpretation, and gives clear definition on what a group is rather than what a group is not.*

2. Split Figure 1 into sub panel a and b can help clean the references in the manuscript.

*Response: We have now incorporated this suggestion in the revised manuscript.*

3. The header in Supplement Table 2 seems to be shifted and causes mismatch. Please fix

*Response: This has been fixed in the revised manuscript.*

4. The author mentioned that splits between B cells and T cells are not significant in the FVB3-mammary example. Is this due to the small number of these cells? Is it possible to try with a smaller near neighbor parameter in Seurat (higher resolution) and see if they will be recovered?

*Response: First, we would like to point out that MultiK did identify B cells and T cells as two groups in the high K space. However, in the second step of MultiK pipeline, SigClust determined the non-significant split between these two groups, and suggested that they were subclasses not classes. Thus, on one level these were called different groups, but at the subclass level. We do feel also, as suggested by the reviewer, that this could be due to the small number of Bcells + Tcells present (168 combined), and thus further methodological testing with such a small sample size would be unlikely to yield robust results. Another possible explanation is that we felt that this could be due to the highly variable gene set used in the analysis. As the gene set was identified from using all cells in the data, it is likely that this gene set contains genes that were just separating the immune cells from other types of cells, and the genes distinguishing between B cells and T cells were not selected. In summary, in large part due to the small number of cells, we do not feel this point merits extensive follow up studies like varying the k-nearest neighbor parameter in Seurat as we do not think the results would be robust and a meaningful evaluation of the number of KNN parameter.*

5. In Figure 3e, the Fibro5 was labeled Neural in 3d. Is Firbo5 mislabeled? Or is Fibro5 more similar to Neural?

*Response: Fibro5 was not mislabeled. Yes, Fibro5 is similar to Neural in the sense that in the low K space, they were assigned to the same cluster as the Neural cells. In Figure 3e (high K space), Fibro5 cluster was annotated according to the top differentially expressed genes for this cluster (see Supplemental Table 4). This is why it is useful to look at the clusters at multiple resolution levels as suggested by MultiK.*

6. In Figure S5, a and b reordered the clusters (x axis) which helped a lot with the visualization. Can we do the same reordering for subplot c and d?

*Response: We don’t understand this comment because we did use a similar ordering scheme in subplot c and d as in subplot a and b. All the clusters (x-axis) in all the subpanels of Figure 5 are ordered by the dataset and separately by white space to help with visualization.*

*In closing, we have addressed the majority of the reviewers’ comments, and feel these responses were helpful to show the advantages, as well as some of the limitations, of our method. We feel the manuscript is improved and hope that it is now found acceptable for publication.*

*Citations:*

*1. van Dijk D, Sharma R, Nainys J, Yim K, Kathail P, Carr AJ, et al. Recovering Gene Interactions from Single-Cell Data Using Data Diffusion. Cell. 2018;*

*2. Huang M, Wang J, Torre E, Dueck H, Shaffer S, Bonasio R, et al. SAVER: Gene expression recovery for single-cell RNA sequencing. Nat Methods. 2018;*

*3. Wang J, Ma A, Chang Y, Gong J, Jiang Y, Qi R, et al. scGNN is a novel graph neural network framework for single-cell RNA-Seq analyses. Nat Commun. 2021;*

*4. Hou W, Ji Z, Ji H, Hicks SC. A systematic evaluation of single-cell RNA-sequencing imputation methods. Genome Biol. 2020;*

*5. Lähnemann D, Köster J, Szczurek E, McCarthy DJ, Hicks SC, Robinson MD, et al. Eleven grand challenges in single-cell data science. Genome Biology. 2020.*

*6. Kiselev VY, Andrews TS, Hemberg M. Challenges in unsupervised clustering of single-cell RNA-seq data. Nat Rev Genet [Internet]. 2019;20(May):273–82. Available from: http://dx.doi.org/10.1038/s41576-018-0088-9*

*7. Liu Y, Hayes DN, Nobel A, Marron JS. Statistical significance of clustering for high-dimension, low-sample size data. J Am Stat Assoc. 2008;*

*8. Huang H, Liu Y, Yuan M, Marron & JS. Statistical Significance of Clustering Using Soft Thresholding. J Comput Graph Stat [Internet]. 2015 [cited 2018 Sep 3];24(4):975–93. Available from: http://amstat.tandfonline.com/action/journalInformation?journalCode=ucgs20*

*9. Schaum N, Karkanias J, Neff NF, May AP, Quake SR, Wyss-Coray T, et al. Single-cell transcriptomics of 20 mouse organs creates a Tabula Muris. Nature. 2018;*

*10. Bach K, Pensa S, Grzelak M, Hadfield J, Adams DJ, Marioni JC, et al. Differentiation dynamics of mammary epithelial cells revealed by single-cell RNA sequencing. Nat Commun. 2017;*

*11. Pal B, Chen Y, Vaillant F, Jamieson P, Gordon L, Rios AC, et al. Construction of developmental lineage relationships in the mouse mammary gland by single-cell RNA profiling. Nat Commun. 2017;*

*12. Giraddi RR, Chung CY, Heinz RE, Balcioglu O, Novotny M, Trejo CL, et al. Single-Cell Transcriptomes Distinguish Stem Cell State Changes and Lineage Specification Programs in Early Mammary Gland Development. Cell Rep. 2018;*

*13. Savas P, Virassamy B, Ye C, Salim A, Mintoff CP, Caramia F, et al. Single-cell profiling of breast cancer T cells reveals a tissue-resident memory subset associated with improved prognosis. Nat Med. 2018;*

*14. Svensson V. Droplet scRNA-seq is not zero-inflated. Nature Biotechnology. 2020.*

*15. Kim T, Zhou X, Chen M. Demystifying “drop-outs” in single cell UMI data. bioRxiv. 2020.*

*16. Wilkerson MD, Hayes DN. ConsensusClusterPlus: A class discovery tool with confidence assessments and item tracking. Bioinformatics. 2010;*

**Second round of review**

**Reviewer 1**

(1) Considering diverse and abundant scRNA-seq data in the public domain, (i) will

the performance of MultiK be affected by different cell numbers and read depth? If yes, how?

Response: MultiK will be affected by these features because it is based on clustering methods, and clustering methods performance can be impacted by different cell numbers and different read depths. We have addressed this issue by focusing on the cell number question, and provided a table (Rebuttal Letter Supplementary Table 1) comparing how different cell numbers affect the number of optimal clusters identified by MultiK using the 3 cell line mixture dataset. As the cell number increases, the number of optimal clusters in the low K space is either 2 or 3 (3 being the true number), and the number of optimal clusters in the high K space has larger effects. It is expected that when the cell number is low, for example, N=150 cells, the smallest group (fibroblast ~10% of cells) may be missed, because there are too few cells to be detected. This analysis suggests around 37 fibroblast cells (N=450 total cells) are sufficient for MultiK to detect this most rare group as a cluster/group in the high K space, and ~50 cells (N=500 total cells) in the low K space. For read depth, we would like to note that the datasets used in our application examples answered this question to some extent. In Table S3, we showed detailed information about each dataset including the sequencing technology. We included datasets generated from both 10X genomics and Smart-seq2 platforms, where the former is known to have lower read depth coverage but higher cell number, and vice versa for the latter. We did not see major differences between the two technologies in terms of the MultiK performance. Having said that, we agree with the reviewer that understanding how different read depths would be an interesting topic itself to study further in the future, but this is more related to a clustering parameter than a MultiK tuning parameter, and thus not further pursued here.

Reviewer: How about large dataset? Is it also capable for rare subset detection in large (>10,000 cells) data? Also, 37 out of 450 cells (8%) can hardly be considered as a rare cell group. A figure or a table is needed to summarize the result of your comparison and include your discussion.

(2) will different sequencing technologies (e.g., 10X genomics and Smart-Seq) have impacts on the result or show any tendencies in MultiK?

Response: As discussed above, in our real dataset application (both mouse mammary gland and human T cell datasets), three of which are Smart-seq2 and the rest are 10X genomics (see Table S3), we did not see major differences from the sequencing technology/platform in the results of MultiK. We say this because we found the same reproducible groups across both platforms in our presented studies. Specifically, in the mouse mammary gland case, we found 6/7 reproducible groups on both 10X and Smart-seq2 platforms in the low K space, and all 8 reproducible groups on both platforms in the high K space (Figure 4, Supplementary Table 16). In the human T cell case, all the reproducible groups were detected by both platforms in both low K and high K space (Figure 5, Figure S7, Supplementary Tables 15, 17). This indicates that our MultiK method is robust to different single cell sequencing technologies.

Reviewer: OK, but still, need a figure or a table to summarize the comparison in your main context or supplementary file.

(3) Will gene imputation in the preprocess step affect (enhance or fail or no significant effect) the result of MultiK results? Is there a preference whether gene imputation should or should not be used, and why?

Response: We believe gene imputation in the preprocessing step will affect MultiK results, however, this is much more of a clustering aspect than a MultiK performance aspect. We note that there are a good number of gene imputation methods developed for scRNAseq data such as MAGIC[1], SAVER[2], and scGNN[3] (which is a novel method that combines gene imputation and clustering using a graph neural network framework). However, evaluating different gene imputation methods is beyond the scope of this paper, and is truly a paper in and of itself. In fact, some studies have addressed this explicitly and for example, Hou et al.[4] performed a systematic evaluation of 18 scRNAseq imputation methods, and concluded that most of the imputation methods failed to improve clustering and trajectory analysis compared to applying no imputation. In addition, most of the imputation methods employ the information from the dataset itself rather than external information, which biases the imputation results[5]. Lastly, the goal of MultiK is to provide objective guidance on the estimation of K. For these reasons, we have chosen not to study gene imputation here but do acknowledge that to impute or not is a very important clustering question.

Reviewer: My point is not asking an evaluation of different imputation methods. Statistical proves are needed for verifying the influences of applying imputation process. I would suggest the author just select one imputation tool and compare the K selection results on at least two datasets (small sample size and large sample size; with and without imputation).

(4) Fundamentally, MultiK is designed for cell clustering, no matter the auto K selection or hierarchical structure it can deliver. Only compare MultiK with Seurat, SC3, and IKAP is not sufficient. The author should add experiments to compare MultiK with more tools, such as BackSPIN, TooManyCells, and scGNN.

Response: We appreciate the reviewer’s suggestion on comparing the performance of MultiK with additional similar methods. First, we would like to point out that MultiK is not a new clustering method, but rather a method that uses an established clustering method (Louvain clustering in Seurat), and its goal is to automatically selects K when given a single data set; the automatic selection of K is the novel contribution. That is why we focused on comparing MultiK to IKAP and SC3, as these methods are designed for determining K in scRNAseq data. We also decided to compare MultiK to the Seurat default resolution parameter, because we wanted to see how MultiK (which considers multiple resolution parameters) performs vs. a single default setting resolution parameter in a widely used package. Second, it is worth noting that the tools suggested by the reviewer here do not provide an objective assessment of what is the data driven optimal K. BackSPIN, TooManyCells, and scGNN were designed for clustering scRNAseq data, especially in the important and challenging case of identifying rare cell populations. BackSPIN uses a divisive biclustering algorithm to identify clusters, and iteratively applies feature selection and cell clustering, which thus may have a tendency to over-partition the data[6]. scGNN uses a graph neural network framework and combines gene imputation and cell clustering, but essentially it is developed for clustering cells. While TooManyCells employs a multi-resolution idea, and provides a tree structure visualization to study the relationship between cell clades, it does not provide an objective determination as to how many clades are statistically significant; instead TooManyCells leaves to the viewer to determine how many groups they feel are present. Because these methods cannot provide an objective assessment of K, it is not possible for us to compare MultiK to these methods. If we do these comparisons, we would have to either rely on the default clustering resolution and/or make a subjective decision on what K to use from these 3 methods, and make a subjective decision about what is the optimal K, which would not provide a fair comparison. This was also exactly why we developed “MultiK”, a tool that provides the user, using the data in hand, a data driven number of "clusters" or "groups"; conversely many clustering tools provide a visualization tool to explore clusters/groups at different resolutions, but then leaves it to the scientist to literally eyeball how many "groups" they think are present. We also now provide more discussion regarding this key point in the Discussion section in the revised manuscript.

Reviewer: The authors need to consider two things. First, for highlighting the optimal K selection function in MultiK, the authors should clearly address the value of MultiK to the research community. How will it help/benefit other clustering methods, especially those tools without a parameter for K selection? Second, I understand that the authors focus on the evaluation of K. However, the value of an optimal K is directly reflected on the cell clustering results. The evaluation of cell clustering is the most intuitive way to assess whether such an optimized K is meaningful or not. One step further, the author claiming the clustering results is more influenced by the clustering methods. Then, the combination of MultiK and different clustering methods should be evaluated. Lastly, x-means is also a way that can determine optimal K and should be compared with MultiK.

(5) The author claimed in the cover letter that the MultiK code is submitted as a

supplementary file, but I could not find it in the system. I could not make any comments on the package/tutorial of this tool. I will suggest uploading the package on GitHub and claim it in the code availability section.

Response: We apologize for this lack of the code, and we note we had significant

difficulties loading the code into the manuscript system. The MultiK code has now been deposited in Github: https://github.com/siyao-liu/MultiK and is publicly available. A short tutorial along with an example is also provided.

Error exists in example code:

1)	the package should be “sigclust” rather than “SigClust”

2)	Error occurred for the example workflow (see details in the attachment)

3)	Where can I download the synthetic data?

(6) Appropriate references are needed to justify the rationale of data synthesis.

Response: The reasons for generating the synthetic datasets from the 3 cell line mixture dataset using our methods are the following:

(i) It is still very difficult to simulate scRNAseq data that mimics real-world data. Thus, we chose to avoid using simulation packages to generate entire scRNAseq

data, because the underlying model is still difficult and requires further investigation. Instead, we took real world data and systematically modified just a small part of this, thus leaving the real-world data structure largely untouched.

(ii) We already have a 3 cell line mixture dataset, in which the cell identities are known, providing a ground truth dataset for us to compare the performance of different methods. A simple way to augment the data is to introduce a synthetic cluster/group of cells by adding in some signals to the original data.

(iii) We chose to modify the gene counts by fitting a Poisson distribution with various Poisson parameters, and this allowed us to see the impact of the signal that we purposely added in the data. It also allowed us to identify the contexts where the synthetic cluster can be identified as a class or a subclass. The reason why we chose to use a Poisson distribution is that multiple recent studies have shown that UMI count scRNAseq data is not significantly zero-inflated and any observed zero-inflation is likely driven by the cell-type heterogeneity and biological variation [14,15]. Particularly, Kim et al.[15] observed that the majority of the genes fit the expected Poisson curve in a homogeneous cell population, and their results strongly suggested that it is unnecessary to model any zero counts using a zero-inflated distribution. Inspired by that, we think it is appropriate to use a simple Poisson distribution to modify a small subset of genes in a subset of luminal cells in our 3 cell line mixture dataset.

We acknowledge that there could be many other ways to generate synthetic scRNAseq data, but this is not the focus of this paper. Our goal here was to generate a set of synthetic datasets with ground truth labels and use them for benchmarking the estimation of cluster number provided by MultiK. Note the synthetic data was just one of multiple ways we benchmarked our method.

Reviewer: It is true that simulation is not the focus of this paper, therefore, why not directly use published methods but designed a new one? Have such a synthetic method or similar one ever used by other studies?

(7) I am not sure what is the necessity to describe the details of the experimental

design of Balbc data. Is it the data newly generated?

Response: Yes, the Balbc scRNA-seq data is new to this paper and thus required an experimental description.

Reviewer: The dataset (GSE165336) is currently not available on GEO. Also, as said previously, the synthetic data is also not available (neither in supplementary or GitHub).

(8) What is the threshold for DEG determination?

Response: For DEG determination, we did group 1 vs. all others, group 2 vs. all others, etc. using the Wilcoxon rank sum test. We used a Bonferroni-corrected p value ≤ 0.05 as the cutoff to identify significant DEGs. Details can be found in the

Methods/Differential expression analysis in the main manuscript.

Reviewer: I am fine with the p-values and adjustment. For single-cell DEG analysis, a threshold of logFC (normally be 0.25) is also needed. Why is the author not applying such criteria?

**Reviewer 2**

I thank the authors’ detailed response. The authors have addressed my comments and concerns. There are still two minor suggestions.

1. As both reviewers asked about the choice of distributions in the synthetic experiment, should the authors add the rationales and references in the “Methods - Synthetic experiment” part of the manuscript?

2. The authors also provided a rich amount of rebuttal supplementary tables, and I believe they are helpful for the broader audiences. Is it possible to integrate these tables into the manuscript and supplementary tables?

**Authors Response**

**Point-by-point responses to the reviewers’ comments:**

*We thank the reviewers for the helpful feedback and suggestions. As was suggested, we have now provided 2 new supplemental figures (Figures S3 and S4) in the manuscript, which address the issues of data simulation and identification of rare cell-types. We also have incorporated the corresponding results and interpretation in the main manuscript. As suggested by Reviewer #1, we also added a paragraph in the Discussion section in the main manuscript addressing the issue of sequencing technology, and we added two more sentences in the Discussion highlighting the value and benefits of MultiK. As suggested by both reviewers, we added the rationale for data generation in our synthetic experiment in the Methods-Synthetic experiment section.*

*We do wish to note, however, that we felt that a few of the new comments from Reviewer #1 were focused on the details of clustering algorithms and clustering methods. We would like to again point out that our work here is not about clustering methods, and that the main point of MultiK is to determine the optimal number(s) of clusters when given a pre-chosen clustering method. In fact, our MultiK method does not propose a novel clustering algorithm, and instead is based on a widely used clustering method (the Louvain clustering implemented in Seurat). The combination of MultiK with different clustering algorithms we feel is beyond the scope of our paper and that the most value to the community is being served through this thorough evaluation using the by far most widely used scRNAseq clustering method. We do feel that we have again addressed most of the reviewers’ comments, that the manuscript is improved, and we now hope that all find the manuscript acceptable for publication.*

REQUESTS AND RESPONSES

Reviewer #1:

(1) Considering diverse and abundant scRNA-seq data in the public domain, (i) will the performance of MultiK be affected by different cell numbers and read depth? If yes, how?

Previous response: MultiK will be affected by these features because it is based on clustering methods, and clustering methods performance can be impacted by different cell numbers and different read depths. We have addressed this issue by focusing on the cell number question, and provided a table (Rebuttal Letter Supplementary Table 1) comparing how different cell numbers affect the number of optimal clusters identified by MultiK using the 3 cell line mixture dataset. As the cell number increases, the number of optimal clusters in the low K space is either 2 or 3 (3 being the true number), and the number of optimal clusters in the high K space has larger effects. It is expected that when the cell number is low, for example, N=150 cells, the smallest group (fibroblast ~10% of cells) may be missed, because there are too few cells to be detected. This analysis suggests around 37 fibroblast cells (N=450 total cells) are sufficient for MultiK to detect this most rare group as a cluster/group in the high K space, and ~50 cells (N=500 total cells) in the low K space. For read depth, we would like to note that the datasets used in our application examples answered this question to some extent. In Table S3, we showed detailed information about each dataset including the sequencing technology. We included datasets generated from both 10X genomics and Smart-seq2 platforms, where the former is known to have lower read depth coverage but higher cell number, and vice versa for the latter. We did not see major differences between the two technologies in terms of the MultiK performance. Having said that, we agree with the reviewer that understanding how different read depths would be an interesting topic itself to study further in the future, but this is more related to a clustering parameter than a MultiK tuning parameter, and thus not further pursued here.

Reviewer: How about large dataset? Is it also capable for rare subset detection in large (>10,000 cells) data? Also, 37 out of 450 cells (8%) can hardly be considered as a rare cell group. A figure or a table is needed to summarize the result of your comparison and include your discussion.

*New response: We now have provided a supplementary figure (Figure S4) and a section named “Identification of rare cell populations” in the Results in the main manuscript to address this question. Specifically, we removed different percentages of the smallest group (fibroblast) in the 3 cell line mixture dataset, and ran MultiK. Supplementary Fig. 4a shows the number of clusters MultiK identified as a function of the percentage of the fibroblast cells in the total number of cells. MultiK accurately identified the rare cell population even when only 0.5% of the cells (N=13) were fibroblast, which demonstrates the robustness of MultiK in detection of rare cell groups. Supplementary Fig. 4b, c shows the MultiK diagnostic plots for 1% and 2% of the total cells as fibroblast cells. In all the cases we have tried, most of them were similar to the results in the 1% case, in which MultiK identified 3 and 7 as optimal Ks, and when K=3, the cell labels from MultiK perfectly matched the true cell labels.*

*Taken together, these analyses suggest that MultiK is robust in identifying both rare and common cell types, and further reveals that MultiK can detect rare cell-types, down to the smallest tested set of 0.5% of prevalence in a data set containing thousands of cells.*

(2) will different sequencing technologies (e.g., 10X genomics and Smart-Seq) have impacts on the result or show any tendencies in MultiK?

Previous response: As discussed above, in our real dataset application (both mouse mammary gland and human T cell datasets), three of which are Smart-seq2 and the rest are 10X genomics (see Table S3), we did not see major differences from the sequencing technology/platform in the results of MultiK. We say this because we found the same reproducible groups across both platforms in our presented studies. Specifically, in the mouse mammary gland case, we found 6/7 reproducible groups on both 10X and Smart-seq2 platforms in the low K space, and all 8 reproducible groups on both platforms in the high K space (Figure 4, Supplementary Table 16). In the human T cell case, all the reproducible groups were detected by both platforms in both low K and high K space (Figure 5, Figure S7, Supplementary Tables 15, 17). This indicates that our MultiK method is robust to different single cell sequencing technologies.

Reviewer: OK, but still, need a figure or a table to summarize the comparison in your main context or supplementary file.

*New response: We now have provided a paragraph summarizing the comparison in the main text. See Discussion section, second paragraph, which reads as follows:*

*Given the diversity and abundance of scRNAseq data available in the public domain, we also observed that MultiK is robust to different single cell sequencing technologies. For example, in our real dataset application (both mouse mammary gland and human T cell datasets), three of which are Smart-seq2 and the rest are 10X genomics (see Table S3), we found the same reproducible groups across both platforms in our presented studies. Specifically, in the mouse mammary gland case, we found 6/7 reproducible groups on both 10X and Smart-seq2 platforms in the low K space, and all 8 reproducible groups on both platforms in the high K space (Fig. 4, Supplementary Table 16). In the human T cell case, all the reproducible groups were detected by both platforms in both low K and high K space (Fig. 5, Supplementary Fig. S7c, d, 9, Supplementary Tables 15, 17).*

(3) Will gene imputation in the preprocess step affect (enhance or fail or no significant effect) the result of MultiK results? Is there a preference whether gene imputation should or should not be used, and why?

Previous response: We believe gene imputation in the preprocessing step will affect MultiK results, however, this is much more of a clustering aspect than a MultiK performance aspect. We note that there are a good number of gene imputation methods developed for scRNAseq data such as MAGIC[1], SAVER[2], and scGNN[3] (which is a novel method that combines gene imputation and clustering using a graph neural network framework). However, evaluating different gene imputation methods is beyond the scope of this paper, and is truly a paper in and of itself. In fact, some studies have addressed this explicitly and for example, Hou et al.[4] performed a systematic evaluation of 18 scRNAseq imputation methods and concluded that most of the imputation methods failed to improve clustering and trajectory analysis compared to applying no imputation. In addition, most of the imputation methods employ the information from the dataset itself rather than external information, which biases the imputation results[5]. Lastly, the goal of MultiK is to provide objective guidance on the estimation of K. For these reasons, we have chosen not to study gene imputation here but do acknowledge that to impute or not is a very important clustering question.

Reviewer: My point is not asking an evaluation of different imputation methods. Statistical proves are needed for verifying the influences of applying imputation process. I would suggest the author just select one imputation tool and compare the K selection results on at least two datasets (small sample size and large sample size; with and without imputation).

*New response: We now have compared MultiK results with and without gene imputation using MAGIC (van Dijk D, Sharma R, Nainys J, Yim K, Kathail P, Carr AJ, et al. Recovering Gene Interactions from Single-Cell Data Using Data Diffusion. Cell. 2018) on two of our datasets: 3 cell line mixture and one of the mouse mammary gland datasets (Rebuttal Table 1 below). We observed that gene imputation very much affected the MultiK results: it significantly increased the number of groups in both low K and high K space. These analyses and results strongly suggest that imputation is a controversial and challenging issue, and that it can have a major impact on all downstream analyses. We note that there are entire papers devoted to this imputation topic including a recent publication by Hou et al. (Hou W, Ji Z, Ji H, Hicks SC. A systematic evaluation of single-cell RNA-sequencing imputation methods. Genome Biol. 2020) where they extensively investigated the impact of imputation on scRNAseq data analysis. In their paper, they evaluated 18 different imputation methods, and concluded that most of the imputation methods failed to improve clustering and trajectory analysis compared to applying no imputation. Hence, we do not feel that we can add beyond what is in the Hou et al. paper on the issue related to whether to impute or not; it would be a very large amount of work to determine how and why MAGIC (or another) imputation method affects clustering and MultiK, and we feel this would all be about imputation and not about testing the merits of MultiK. We again highlight that gene imputation is not directly relevant to the focus of our paper, nor did we ever use it or suggest it to be used. Given the outstanding work done in the Hou et al. paper that extensively evaluated the effect of gene imputation, we feel that imputation is an interesting open question, but not related to what MultiK is proposed to do and is beyond the scope of our paper.*

(4) Fundamentally, MultiK is designed for cell clustering, no matter the auto K selection or hierarchical structure it can deliver. Only compare MultiK with Seurat, SC3, and IKAP is not sufficient. The author should add experiments to compare MultiK with more tools, such as BackSPIN, TooManyCells, and scGNN.

Previous response: We appreciate the reviewer’s suggestion on comparing the performance of MultiK with additional similar methods. First, we would like to point out that MultiK is not a new clustering method, but rather a method that uses an established clustering method (Louvain clustering in Seurat), and its goal is to automatically selects K when given a single data set; the automatic selection of K is the novel contribution. That is why we focused on comparing MultiK to IKAP and SC3, as these methods are designed for determining K in scRNAseq data. We also decided to compare MultiK to the Seurat default resolution parameter, because we wanted to see how MultiK (which considers multiple resolution parameters) performs vs. a single default setting resolution parameter in a widely used package. Second, it is worth noting that the tools suggested by the reviewer here do not provide an objective assessment of what is the data driven optimal K. BackSPIN, TooManyCells, and scGNN were designed for clustering scRNAseq data, especially in the important and challenging case of identifying rare cell populations. BackSPIN uses a divisive biclustering algorithm to identify clusters, and iteratively applies feature selection and cell clustering, which thus may have a tendency to over-partition the data[6]. scGNN uses a graph neural network framework and combines gene imputation and cell clustering, but essentially it is developed for clustering cells. While TooManyCells employs a multi-resolution idea, and provides a tree structure visualization to study the relationship between cell clades, it does not provide an objective determination as to how many clades are statistically significant; instead TooManyCells leaves to the viewer to determine how many groups they feel are present. Because these methods cannot provide an objective assessment of K, it is not possible for us to compare MultiK to these methods. If we do these comparisons, we would have to either rely on the default clustering resolution and/or make a subjective decision on what K to use from these 3 methods, and make a subjective decision about what is the optimal K, which would not provide a fair comparison. This was also exactly why we developed “MultiK”, a tool that provides the user, using the data in hand, a data driven number of "clusters" or "groups"; conversely many clustering tools provide a visualization tool to explore clusters/groups at different resolutions, but then leaves it to the scientist to literally eyeball how many "groups" they think are present. We also now provide more discussion regarding this key point in the Discussion section in the revised manuscript.

Reviewer: The authors need to consider two things. First, for highlighting the optimal K selection function in MultiK, the authors should clearly address the value of MultiK to the research community. How will it help/benefit other clustering methods, especially those tools without a parameter for K selection? Second, I understand that the authors focus on the evaluation of K. However, the value of an optimal K is directly reflected on the cell clustering results. The evaluation of cell clustering is the most intuitive way to assess whether such an optimized K is meaningful or not. One step further, the author claiming the clustering results is more influenced by the clustering methods. Then, the combination of MultiK and different clustering methods should be evaluated. Lastly, x-means is also a way that can determine optimal K and should be compared with MultiK.

*New response: There were many questions posed here, and forgive us if not all are addressed. First point is we now have added three sentences in the Discussion, third paragraph in the main manuscript, highlighting the value and benefits of applying MultiK, which reads as follows: “Importantly, MultiK benefits most clustering methods because those need an input value of K to even begin a clustering analysis. In these cases, we recommend running MultiK first, which will give a rigorous choice of K for use as input to any preferred clustering method of interest that could be run next. For methods that do not need an input choice of K, but which do not provide outputs on the optimal K, MultiK provides such a data driven suggestion for optimal K in both the low-resolution space and high-resolution space.”*

*Second point is for the evaluation of MultiK with additional clustering methods, which we feel is far beyond the scope of this paper. Our goal is not about evaluating different clustering methods, nor is it to determine if different clustering methods agree with each other or not, but instead is focused on development of a method that provides objective assessment of K when using a standard and widely used scRNAseq clustering approach. There are just too many other methods to possibly try, and then it would evolve into comparisons of agreements/disagreements between clustering methods, which would not directly test the value of MultiK.*

*Lastly, we performed a literature search on X-means clustering and it is a very sparely used method. This method was first proposed two decades ago, and to the best of our knowledge, X-means has not been applied to scRNAseq data (nor possibly even applied in bulk tumor gene expression data). Because the world of clustering is extremely large, it is impractical to compare our chosen clustering method against multiple other methods that have ever even been proposed for choosing K. As stated above, we feel evaluation of different clustering methods is a worthy topic but not for this paper, and that to thoroughly evaluate which clustering method is best (which is what we would need to end up doing since we already know different clustering methods would likely give different optimal Ks) is a paper’s worth of work in and of itself; we therefore feel this is beyond the scope of what is necessary to demonstrate the value of MultiK.*

(5) The author claimed in the cover letter that the MultiK code is submitted as a supplementary file, but I could not find it in the system. I could not make any comments on the package/tutorial of this tool. I will suggest uploading the package on GitHub and claim it in the code availability section.

Previous response: We apologize for this lack of the code, and we note we had significant difficulties loading the code into the manuscript system. The MultiK code has now been deposited in Github: https://github.com/siyao-liu/MultiK and is publicly available. A short tutorial along with an example is also provided.

Reviewer: Error exists in example code:1) the package should be “sigclust” rather than “SigClust”2) Error occurred for the example workflow (see details in the attachment)3) Where can I download the synthetic data?

*New response: (1) The package name is “sigclust” but the method name is “SigClust”.*

*(2) The error was caused by the example data size. Now we have provided a larger dataset for demonstration that fixes the error.*

*(3) The synthetic data are now available on Github: https://github.com/siyao-liu/MultiK/tree/main/analysis/synthetic_datasets*

(6) Appropriate references are needed to justify the rationale of data synthesis.

Previous response: The reasons for generating the synthetic datasets from the 3 cell line mixture dataset using our methods are the following:(i) It is still very difficult to simulate scRNAseq data that mimics real-world data. Thus, we chose to avoid using simulation packages to generate entire scRNAseq data, because the underlying model is still difficult and requires further investigation. Instead, we took real world data and systematically modified just a small part of this, thus leaving the real-world data structure largely untouched. (ii) We already have a 3 cell line mixture dataset, in which the cell identities are known, providing a ground truth dataset for us to compare the performance of different methods. A simple way to augment the data is to introduce a synthetic cluster/group of cells by adding in some signals to the original data. (iii) We chose to modify the gene counts by fitting a Poisson distribution with various Poisson parameters, and this allowed us to see the impact of the signal that we purposely added in the data. It also allowed us to identify the contexts where the synthetic cluster can be identified as a class or a subclass. The reason why we chose to use a Poisson distribution is that multiple recent studies have shown that UMI count scRNAseq data is not significantly zero-inflated and any observed zero-inflation is likely driven by the cell-type heterogeneity and biological variation [14,15]. Particularly, Kim et al.[15] observed that the majority of the genes fit the expected Poisson curve in a homogeneous cell population, and their results strongly suggested that it is unnecessary to model any zero counts using a zero-inflated distribution. Inspired by that, we think it is appropriate to use a simple Poisson distribution to modify a small subset of genes in a subset of luminal cells in our 3 cell line mixture dataset. We acknowledge that there could be many other ways to generate synthetic scRNAseq data, but this is not the focus of this paper. Our goal here was to generate a set of synthetic datasets with ground truth labels and use them for benchmarking the estimation of cluster number provided by MultiK. Note the synthetic data was just one of multiple ways we benchmarked our method.

Reviewer: It is true that simulation is not the focus of this paper, therefore, why not directly use published methods but designed a new one? Have such a synthetic method or similar one ever used by other studies?

*New response: We acknowledge that our synthetic data method has not been used by others, but we feel that it targets the central issue here, that it gives clear definitive results, and we stand by these results. First, we now have added a paragraph in the Methods-Synthetic Experiment section, explaining the rationale of data generation from our synthetic experiment. Second and in response to this comment, we have now provided results from using a different simulation method, see Supplementary Figure 3, which again demonstrated the ability of MultiK to find known groups. We added Results-Synthetic dataset experiments section in the main manuscript showing these simulation results, which reads as follows:*

*For the second set of synthetic experiments, we also simulated 3 scRNAseq datasets with known groups using Splatter package[29], and tested the performance of MultiK. Each dataset contained 5,000 genes or 1,000 cells. The first dataset had 2 equal groups (with 500 cells in each group); the second dataset had 3 unequal groups (with group probabilities 0.1, 0.3, and 0.6 and 1,000 number of total cells); the third dataset had 5 equal groups (with 200 cells in each group). Visual inspection of PCA projections showed distinct separation of the simulated groups (left column in Supplementary Fig. 3), except in the 5 equal group data set where 3 of the 5 groups were clustered together, which could be due to the number of PC projections being displayed here. As expected, in each dataset, MultiK successfully identified the expected number of groups in the low K space, and the cell labels perfectly matched the group labels (right columns in Supplementary Fig. 3).*

(7) I am not sure what is the necessity to describe the details of the experimental design of Balbc data. Is it the data newly generated?

Previous response: Yes, the Balbc scRNA-seq data is new to this paper and thus required an experimental description.

Reviewer: The dataset (GSE165336) is currently not available on GEO. Also, as said previously, the synthetic data is also not available (neither in supplementary or GitHub).

*New response: GSE165336 is in GEO and are now made public even before publication to accommodate this reviewers request. The synthetic data are now available on Github: https://github.com/siyao-liu/MultiK.*

(8) What is the threshold for DEG determination?

Previous response: For DEG determination, we did group 1 vs. all others, group 2 vs. all others, etc. using the Wilcoxon rank sum test. We used a Bonferroni-corrected p value ≤ 0.05 as the cutoff to identify significant DEGs. Details can be found in the Methods/Differential expression analysis in the main manuscript.

Reviewer: I am fine with the p-values and adjustment. For single-cell DEG analysis, a threshold of logFC (normally be 0.25) is also needed. Why is the author not applying such criteria?

*New response: We did not apply such a fold-change criteria because we feel it is not necessary. We are interested in genes that show significant differential expression, including potentially both low fold change or high fold change, noting there is likely important biology in both such sets of genes. That is why we did not apply such a fold-change criteria.*

Reviewer #2: I thank the authors’ detailed response. The authors have addressed my comments and concerns. There are still two minor suggestions.

1. As both reviewers asked about the choice of distributions in the synthetic experiment, should the authors add the rationales and references in the “Methods - Synthetic experiment” part of the manuscript?

*Response: As said above, we now have provided a paragraph in the Methods-Synthetic Experiment section, explaining the rationale of data generation from our synthetic experiment, and provided a second synthetic data generation analysis as well.*

2. The authors also provided a rich amount of rebuttal supplementary tables, and I believe they are helpful for the broader audiences. Is it possible to integrate these tables into the manuscript and supplementary tables?

*Response: As said at the beginning of this rebuttal letter, we now have provided 2 additional supplemental figures (Figure S3 and S4) to address some of the issues raised by both reviewers.*

*In closing, we have addressed most of the reviewer’s comments, and feel these responses were helpful to show the advantages and benefits of our method. We feel the manuscript is improved and hope that it is now found acceptable for publication.*

**Third round of review**

**Reviewer 1**

Thanks for fixing all my concerns.
